# Molecular Evolutionary Consequences of Niche Restriction in *Francisella tularensis*, a Facultative Intracellular Pathogen

**DOI:** 10.1371/journal.ppat.1000472

**Published:** 2009-06-12

**Authors:** Pär Larsson, Daniel Elfsmark, Kerstin Svensson, Per Wikström, Mats Forsman, Thomas Brettin, Paul Keim, Anders Johansson

**Affiliations:** 1 Division of CBRN Defence and Security, Swedish Defence Research Agency, Umeå, Sweden; 2 Department of Clinical Microbiology, Infectious Diseases and Bacteriology, Umeå University, Umeå, Sweden; 3 Joint Genome Institute, Los Alamos National Laboratories, Los Alamos, New Mexico, United States of America; 4 Northern Arizona University, Flagstaff, Arizona, United States of America; 5 Translational Genomics Research Institute, Phoenix, Arizona, United States of America; University of Toronto, Canada

## Abstract

*Francisella tularensis* is a potent mammalian pathogen well adapted to intracellular habitats, whereas *F. novicida* and *F. philomiragia* are less virulent in mammals and appear to have less specialized lifecycles. We explored adaptations within the genus that may be linked to increased host association, as follows. First, we determined the genome sequence of *F. tularensis* subsp. *mediasiatica*, the only subspecies that had not been previously sequenced. This genome, and those of 12 other *F. tularensis* isolates, were then compared to the genomes of *F. novicida* (three isolates) and *F. philomiragia* (one isolate). Signs of homologous recombination were found in ∼19.2% of *F. novicida* and *F. philomiragia* genes, but none among *F. tularensis* genomes. In addition, random insertions of insertion sequence elements appear to have provided raw materials for secondary adaptive mutations in *F. tularensis*, e.g. for duplication of the *Francisella* Pathogenicity Island and multiplication of a putative glycosyl transferase gene. Further, the five major genetic branches of *F. tularensis* seem to have converged along independent routes towards a common gene set via independent losses of gene functions. Our observations suggest that despite an average nucleotide identity of >97%, *F. tularensis* and *F. novicida* have evolved as two distinct population lineages, the former characterized by clonal structure with weak purifying selection, the latter by more frequent recombination and strong purifying selection. *F. tularensis* and *F. novicida* could be considered the same bacterial species, given their high similarity, but based on the evolutionary analyses described in this work we propose retaining separate species names.

## Author Summary

The intracellular bacterium *Francisella tularensis* causes the disease tularemia in various mammals, including humans, and is highly infectious (so infectious that highly virulent forms of the pathogen were developed as biological aerosol weapons during the Cold War). Little is known about where *F. tularensis* resides in nature and how it evolved but, intriguingly, closely related *Francisella* bacteria are less dangerous. Therefore, we have explored the evolutionary events that shaped *F. tularensis* by analyzing 17 *Francisella* genome sequences. Its evolution appears to have involved many losses of metabolic functions and random mutations, with little exchange of genetic material among *F. tularensis* strains. Furthermore, increased host association appears to have irreversibly separated *F. tularensis* populations from other populations of *Francisella* bacteria. This study provides new information on the processes whereby relatively harmless *Francisella* bacteria evolved into aggressive invaders of mammalian cells. Our findings support previous proposals that identification of distinct population lineages provides meaningful species boundaries among bacteria.

## Introduction

*Francisella tularensis* is probably best known, and most feared, for its potential as a bacterial biological weapon [1]. As such this pathogen was grown and stockpiled in large quantities during the Cold War by both the U.S. and the former Soviet Union. Today, the most virulent *Francisella* strains are among the six biological agents considered to pose the greatest potential public health threats if used by terrorists [2]. Strains of *F. tularensis* subsp. *tularensis* can be lethal to humans, doses as low as 10–25 bacteria can be infective, and transmission can occur via skin inoculation or aerosols [1].

However, in addition to its potentially destructive applications, the genus *Francisella* provides interesting models for studying processes whereby quite harmless environmental bacteria may become transformed into host-restricted and highly virulent human pathogens. In this respect *Francisella* bacteria appear to be in a state previously attributed to several other human pathogens (e.g. *Shigella flexneri*, *Salmonella enterica* serovar Typhi and *Yersinia pestis* [3]) that are in intermediate stages of a genome-erosion process associated with early stages of host-restriction. *Francisella* strains are attractive (as model organisms) since they span a broad range of functional diversity, from strains with high metabolic capacity that are easily grown on artificial media and exhibit low disease potential in humans, to specialized, highly pathogenic bacteria with reduced metabolic capacities that (hence) require very rich culture media [4].

Besides *F. tularensis*, the genus *Francisella* includes two accepted species, *F. philomiragia* and *F. novicida*, both of which are isolated from environmental samples [5],[6]. In contrast to *F. tularensis*, *F. philomiragia* and *F. novicida* are metabolically competent and thus much less fastidious in their growth requirements. They are only rarely human pathogens, the only diseased individuals they have been isolated from were nearly drowned or suffered from a weakened immune system [4].

*F. tularensis* is the cause of tularemia and is characterized as a facultative intracellular pathogen. Tularemia is a typical zoonosis; it is frequently arthropod vector-borne, transmissible to humans, and its usual host is a non-human animal [7]. However, the term “usual host” is somewhat arbitrary for *F. tularensis*; according to a recent review known susceptible species include 190 mammals, 88 invertebrates, 23 birds, and three amphibians [8].

At present, three *F. tularensis* subspecies are accepted and known to cause infections in humans [9]. Two of these are clinically important: subsp. *tularensis* (type A) is found exclusively in North America and may cause severe and life-threatening infections, while subsp. *holarctica* (type B) occurs throughout the Northern Hemisphere and is associated with milder clinical symptoms [10]. Both subspp. have been implicated in the production of biological weapons, although the use of subsp. *tularensis* as a weapon would be more serious. Two major subpopulations among type A strains are designated A1 and A2, respectively [11]. The third *F. tularensis* subspecies, subsp. *mediasiatica*, has only been isolated in areas of central Asia and is reported to exhibit comparable virulence to that of the *holarctica* subspecies [12],[13]. In a rabbit model, strains of *F. tularensis* subspp. *holarctica* and *mediasiatica* kill at a dose of >10^6^ microbial cells, while a lethal dose of subsp. *tularensis*, is 1–10 cells [14]. However, previous studies indicate that subsp. *mediasiatica* is, in evolutionary terms, the closest neighbor to subsp. *tularensis* [15]–[17]. We hypothesized, therefore, that comparing the genome of subsp. *tularensis* to that of subsp. *mediasiatica*, which exhibits lower disease potential for humans, would be valuable for determining factors that cause high pathogenicity in subsp. *tularensis*. Sequencing a genome of a subsp. *mediasiatica* strain would additionally allow for multiple genome comparisons to understand the evolution of human pathogenic strains of genus *Francisella*.

## Results

### The genome of *F. tularensis* subsp. *mediasiatica*

Previously, genome sequences have been reported for two of three recognized *F. tularensis* subspecies. The third subspecies, *F. tularensis* subsp. *mediasiatica*, has been isolated only in dry areas of Central Asia. We describe here the genome of strain FSC147, isolated in the Alma-Ata region of Kazakhstan in 1965, from the rodent species *Meriones meridianus* (Midday gerbil). The genome is composed of a single circular 1,893,886 bp chromosome with an average G+C content of 32.25% (Table 1). It contains 1,470 predicted protein-coding genes and 263 pseudogenes. As in previously characterized representatives of subspp. *tularensis* and *holarctica* [18]–[21], we found three rRNA operons, 38 tRNA genes with 30 anticodons for 20 amino acids, and seven types of insertion sequence (IS) elements. ISFtu1 and ISFtu2 were the most abundant elements, with 59 and 17 copies, respectively (Table 1).

**Table 1:** General features of seven completed *Francisella* genomes.

Property	Species and strain designation (major genetic group)						
	*F. novicida* U112	*F. tularensis* subsp. *tularensis* (A1) SCHUS4	*F. tularensis* subsp. *tularensis* (A2) WY-96	*F. tularensis* subsp. *mediasiatica* FSC147	*F. tularensis* subsp. *holarctica* LVS	*F. tularensis* subsp. *holarctica* OSU18	*F. tularensis* subsp. *holarctica* FTA
Chromosome size (bp)	1,910,036	1,892,819	1,898,476	1,893,886	1,895,998	1,895,727	1,890,909
G+C content (%)	32,47	32,25	32,26	32,25	32,15	32,15	32,16
ISFtu1^a^	1	53	53	59	61	63	61
ISFtu2	17	16	18	17	44	42	44
ISFtu3	4	4	4	4	4	4	4
ISFtu4	1	1	1	1	1	1	1
ISFtu5	0	1	1	1	1	1	1
ISFtu6	2	2	2	2	2	2	2
ISSod13	0	1	1	1	1	1	1

a Abbreviation for Insertion Sequence element, *F. tularensis* 1.

b Abbreviation for Insertion Sequence element, *Shewanella oneidensis* 13.

All predicted genes in *F. tularensis* subsp. *mediasiatica* strain FSC147 were found either in the *tularensis* subspecies (SCHU S4) or *F. novicida* (strain U112). Rhomer et al. suggested that six genes predicted to be functional in an 11.1 kb region (loci FTT1066-FTT1073), together with three other genes (loci FTT1308c, FTT1580c, FTT1791), may promote the high virulence of subsp. *tularensis* since these genes appeared to be specific to strain SCHU S4 when compared with *F. novicida* strain U112 and two strains of *F. tularensis* subsp. *holarctica* [22]. However, all open reading frames in the 11.1 kb region appear to be present and intact in the *mediasiatica* genome of strain FSC147, indicating that these genes by themselves cannot explain the different degrees of virulence. In addition, FTT1308c is inactivated by a frameshift mutation in *mediasiatica* and is not present in *F. tularensis* subsp. *holarctica*, but the intact gene is present in the *F. novicida* isolate 3548, suggesting that this gene is probably not responsible for the high virulence of subsp. *tularensis* SCHU S4 either. Furthermore, FTT1580c encodes a protein of unknown function and contains a frameshift mutation close to the N-terminal part in *F. tularensis* subsp. *mediasiatica* FSC147 that could disrupt the function of the encoded protein. A role for FTT1580c in enhancing virulence is also disputed since the ortholog is intact in *F. tularensis* subsp. *holarctica* FSC022, which exhibits low virulence. Finally, the hypothetical protein referred to as FTT1791 has a nonsense mutation in *mediasiatica*. This gene is also missing in the isolate WY-96 of the type A2 clade of subspecies *tularensis*. Thus, if the isolate WY-96 possesses the high level of virulence ascribed to other strains of subsp. *tularensis* this would exclude FTT1791 as a likely cause.

### Evolutionary relationships and average nucleotide identities of multiple *Francisella* genomes

**Figure 1:**
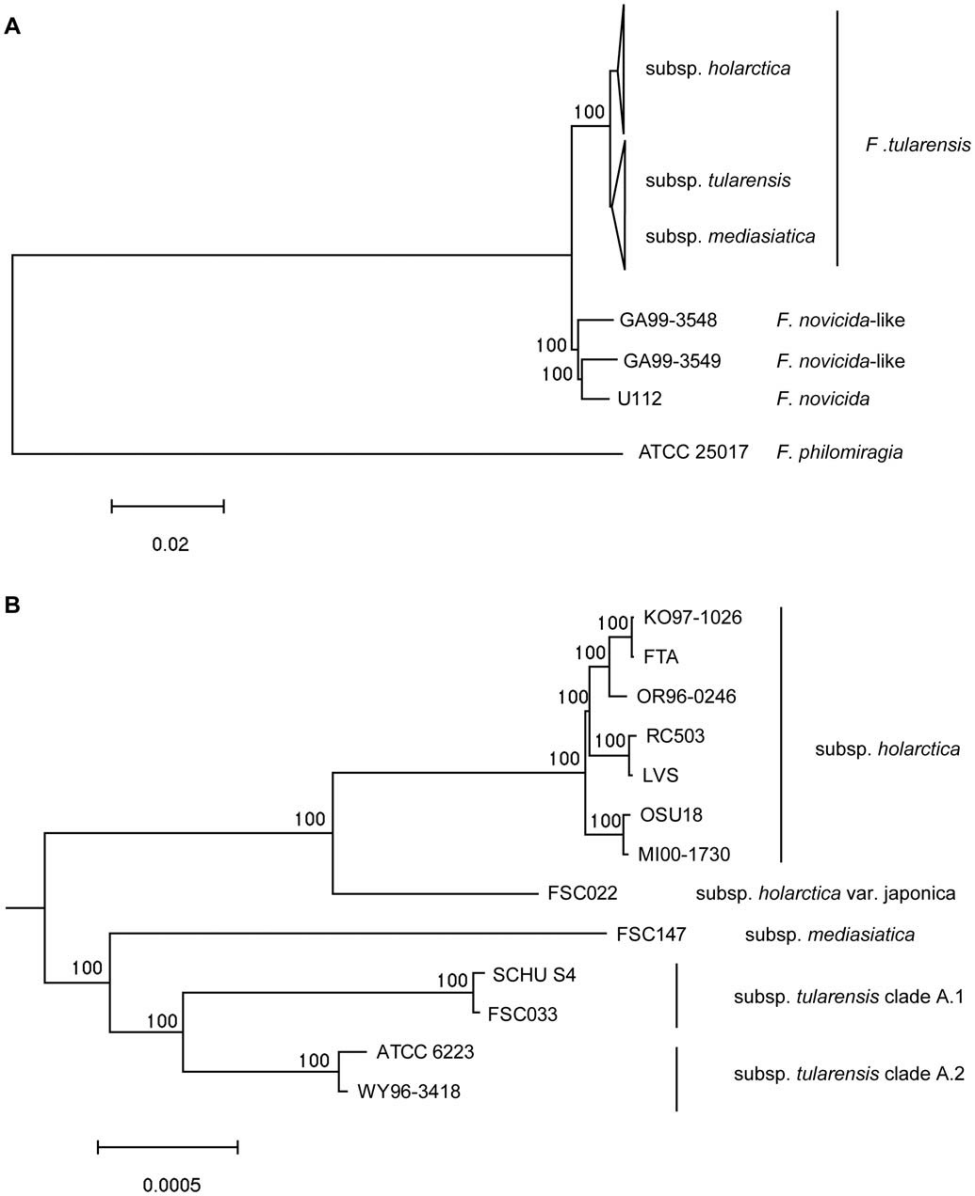
Whole genome phylogeny among 17 *Francisella* strains based on 1,104,129 aligned nucleotide positions. Panel (A) depicts relationships among major clades within the *Francisella* genus and panel (B) relationships within the species *F. tularensis*. The evolutionary tree was inferred using the Neighbor-Joining method. Bootstrap support values (500 replicates) are shown next to branches. Scale bars indicate the number of base substitutions per site.

Using 1,104,129 aligned genomic nucleotide sites, we estimated a whole-genome phylogeny (Figure 1) for one *F. philomiragia*, three *F. novicida* or “*novicida*-like”, and 13 *F. tularensis* strains (Table S1). Both the neighbor-joining and maximum likelihood methods identified an identical topology, with maximal support for all nodes in bootstrap analyses. The tree was consistent with the previous assumption that *F. tularensis* and *F. novicida* constitute sister groups, and confirmed that *F. tularensis* subspp. *mediasiatica*, *holarctica*, and *tularensis* form a monophyletic group [7],[16]. The data extend previous findings by demonstrating the monophyly of *F. novicida* and the *novicida*-like isolates that were included.

Substantial differences in branch lengths were found in the phylogenetic reconstructions (Figure 1), indicating that historic mutation rates have differed among *F. tularensis* genetic lineages. Exemplifying the extremes, mutations in the *F. tularensis* subsp. *tularensis* A2 lineage have occurred much less frequently than in the subsp. *holarctica* lineage (Figure 1). Increased rates of mutation may occur because some DNA repair functions are lost; therefore we scrutinized corresponding genes in the *F. philomiragia*, *F. novicida* and *F. tularensis* genomes. Among 37 potential DNA repair genes we found only one possible candidate, a deoxyribodipyrimidine photolyase gene, *phrB*, that appears to be functional in WY-96, but disrupted in other strains (Table S2). The Phr protein enhances repair of UV-light induced DNA damage and is lacking in many bacterial species because they live in environments where they are not exposed to UV light [23].

The *F. novicida*, *novicida*-like isolates, and *F. tularensis* isolates were found to display considerable overall genetic relatedness. Pairwise analyses of average nucleotide identities (ANI) [24] demonstrated that all combinations had ANI values ≥97.7%. If only isolates of *F. tularensis* were considered, ANI values ≥99.2% (Table S3) were obtained, thus demonstrating a striking level of genetic monomorphism within this monophyletic group of strains, which includes three separate subspecies: *tularensis*, *mediasiatica*, and *holarctica*. In contrast, comparisons that included the *F. philomiragia* isolate ATCC 25017 provided significantly lower ANI estimates, ranging between 80.6% and 81.2%.

### Recombination in *F. philomiragia* and *F. novicida*, and its absence in *F. tularensis*

The presence and extent of recombination in *Francisella* was rigorously investigated using several strategies: visual exploration of genomic data, estimation of recombination and mutation parameters using the ClonalFrame [25] and *R_m_* [26] methods, and estimation of the proportions of genes potentially affected by recombination using a combination of the MaxChi2 [27] and Phi [28] tests. To assess the possibility that recombination has occurred among the highly similar *F. tularensis* lineages, we also used the Maynard Smith and Smith [29] homoplasy test.

**Figure 2:**
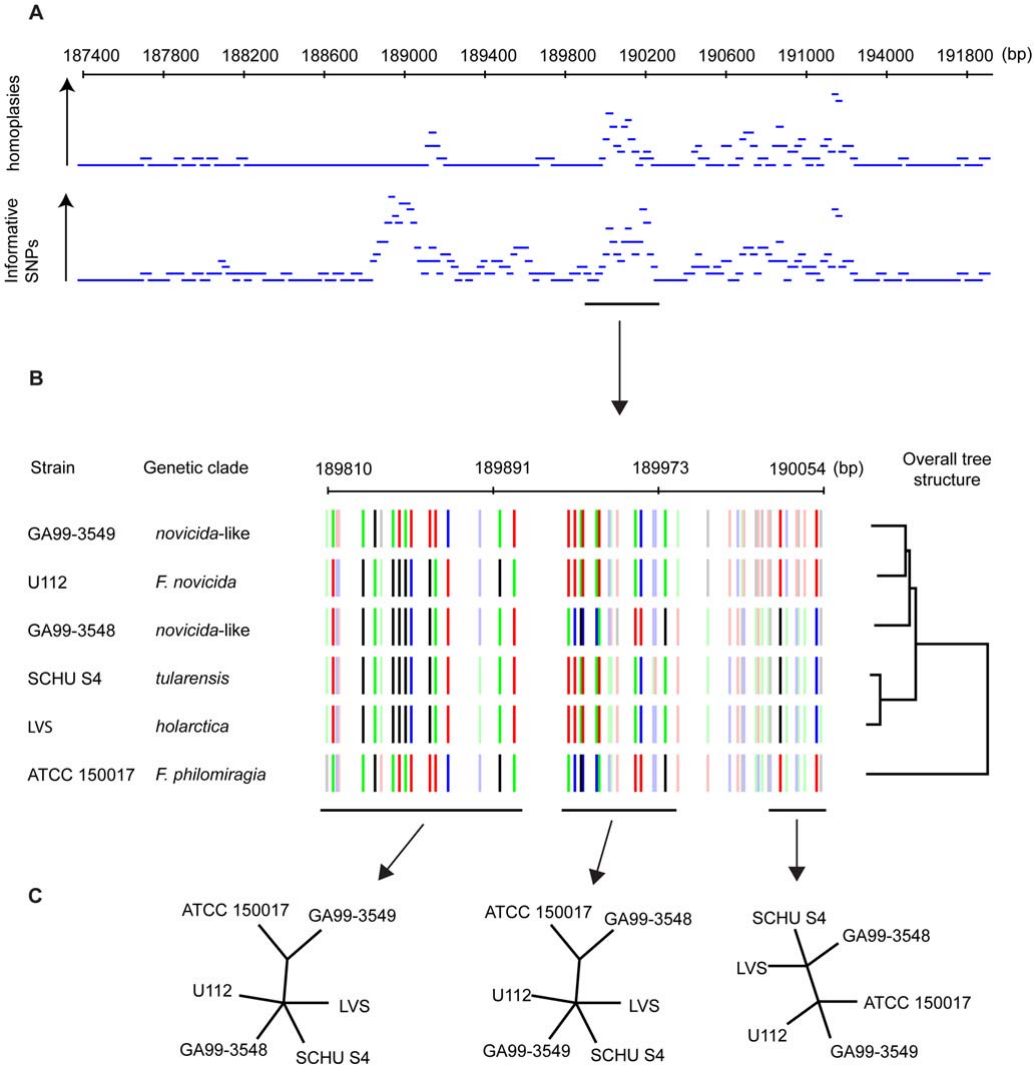
Illustration of one of many recombination events detected in basal parts of the *Francisella* phylogeny. (A) shows that an accumulation of phylogenetically informative SNPs is correlated with homoplastic SNPs. (B) is a magnification illustrating that the SNP patterns are incongruent with the overall whole genome SNP tree. (C) shows that the patterns indicate multiple recombination events in the region, as seen from several conflicting tree topologies.

Visual exploration of colour-coded nucleotide plots revealed indications of numerous past recombination events among metabolically independent *Francisella* lineages (the *F. philomiragia*, *F. novicida*, and ancestral *F. tularensis* lineages; Figure 2). Abundant tracts containing incongruent sites were found, and loci with increased numbers of informative sites, suggestive of recombination between sister lineages. However, we found no evidence to support the occurrence of past recombination between the metabolically independent *Francisella* and any modern lineage of *F. tularensis*. The few genomic regions potentially indicative of such events could be dismissed after close examination as being due to other evolutionary events, such as gene conversion (recombination within the genome) or incomplete lineage sorting (differential loss of previously duplicated genes).

The impact of recombination on *F. philomiragia*, *F. novicida*, and the ancestral branch of *F. tularensis* was further quantified by applying the Clonalframe algorithm to whole genomes and, for comparison, by analyzing five collinear genomic regions to estimate minimum numbers of recombination events and segregating sites. Clonalframe analysis of 1,527,362 sites estimated the 95% credibility region of rho/theta, the ratio of absolute numbers of recombination and mutation events, to be 0.079–0.089. This indicates that mutation, despite an abundance of recombination footprints, has clearly been the predominant evolutionary process. The 95% credibility region of r/m, indicating the probability of recombination versus mutation per individual nucleotide site, was 0.78–0.89, illustrating that the impact of recombination on genetic diversity has been significant.

Furthermore, minimum numbers of recombination events (*R_m_*) were estimated, as described by Hudson and Kaplan [26], for segregating sites in five 75-kb locally collinear sequence blocks. This analysis suggested a rate of recombination an order of magnitude lower than that of mutation (Table 2). However, since *R_m_* represents a lower bound and ClonalFrame models only recombination “imports”, both methods likely underestimate the true number of recombination events

**Table 2:** Minimal number of recombination events in basal parts of the *Francisella* phylogeny.

Genomic alignments^a^	Position in LVS^b^	Minimal number of recombination, *R_m_*	Segregating sites, *S*
Region 1	1,693,555–1,770,700	98	1551
Region 2	1,477,613–1,556,588	149	2018
Region 3	732,862–822,030	135	1760
Region 4	822031–904616	137	3019
Region 5	904,617–991,393	107	1889

a Five local collinear blocks with a total length of 75 kb were analyzed.

b The intervals refer to GenBank accession no. AM233362.1.

We also assessed the proportions of genes affected by recombination in the environmental lineages by a combined analysis in which individual gene alignments were tested by the maximum chi-squared method [27] and the Phi method [28]. This combination of methods was used to increase sensitivity, since they detect different, complementary recombination signals. The maximum chi-squared method and the phi method indicated 223/1251 and 101/1251 genes to have been affected by recombination, respectively (**p<0.01). Using either method, significant indications of recombination were obtained for19.2% of the genes (240/1251) tested.

Because of the limited diversity of *F. tularensis*, few tests can be used to assess recombination within this species. However, in nucleotide alignments representing 13 *F. tularensis* genomes 21 apparent homoplasies were recorded. Therefore we applied Maynard Smith and Smith's homoplasy test [29], which assesses whether there is an excess of homoplasies, compared to expected numbers derived by mutation in the absence of recombination. The null hypothesis of clonality was not rejected using any reasonable estimate of *S_e_*. Thus, we found no evidence to support the hypothesis that recombination has occurred among *F. tularensis* lineages.

### Different action of natural selection in different *Francisella* populations

**Figure 3:**
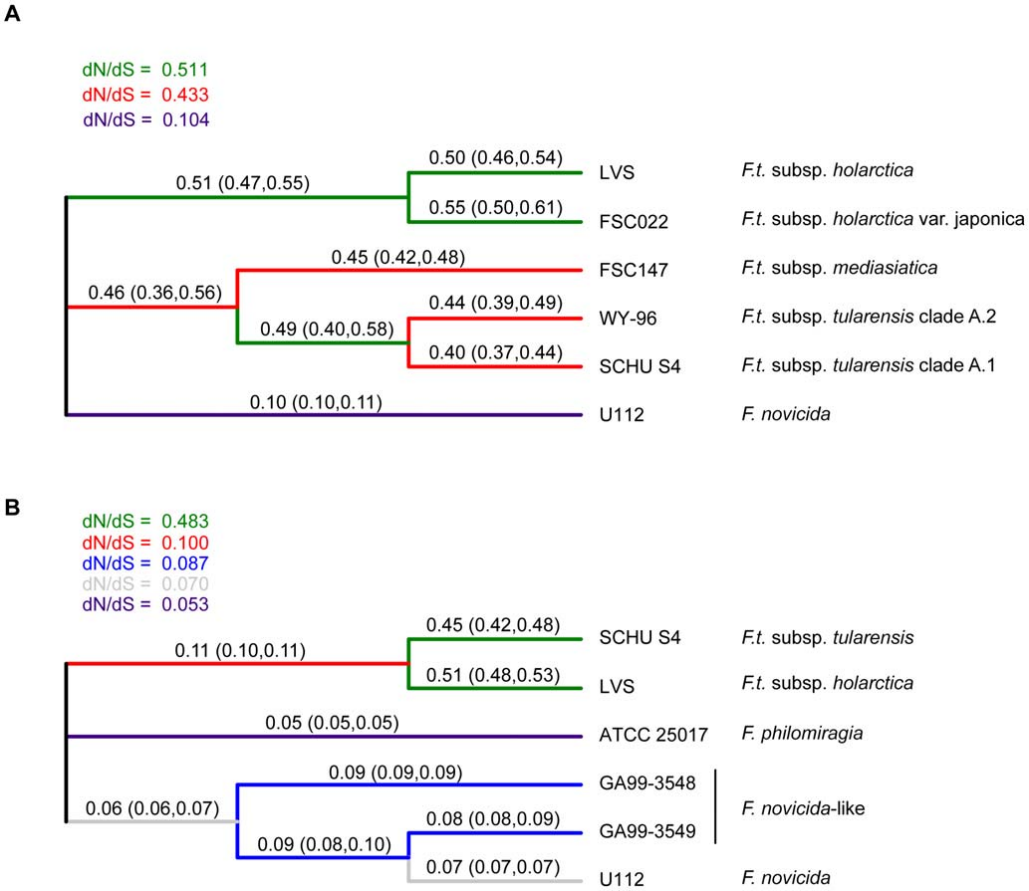
Likelihood estimates of dN/dS ratios across the *Francisella* phylogeny obtained using the software package Hyphy. The analysis indicates statistically significant differences in dN/dS values for different tree branches. Different colors represent the maximum number of rate classes estimated from the data using the Akaike information criterion (AIC) with a genetic algorithm. Values on the branches represent local optima, with confidence intervals in brackets. To optimize the evolutionary models comparisons were separately optimized for the phylogeny of (A) *F. tularensis* strains, and (B) the *Francisella* genus, including *F. philomiragia* and *F. novicida*.

Measuring dN/dS might provide some information on how ecology constrained the evolution of *Francisella* at population levels. We calculated by maximum likelihood methodology [30] lineage-specific dN/dS estimates of selection pressures across the *Francisella* phylogeny (Figure 3), taking care to avoid overfitting by using information-rich genome-wide datasets of aligned codons, and employing a genetic algorithm that optimizes model complexity [30]. To increase the accuracy of our calculations, we focused on *F. tularensis* representatives in one analysis, and on *F. novicida* and *F. philomiragia* in a separate analysis. Overall, our results revealed distinct differences between the environmental lineages (*F. novicida*, *F. philomiragia*) and *F. tularensis*, since we found high dN/dS ratios for all *F. tularensis* branches, and considerably lower ratios for the environmental lineages (Figure 3). Thus, the data indicates that slightly deleterious mutations have been inefficiently removed following the formation of the *F. tularensis* species. This lends support to the hypotheses that effective population sizes of these strains have been low, and successful recombination events among them have been rare or non-existent, consistent with evolution in a favorable intracellular environment. However, there were statistically significant dN/dS differences among *F. tularensis* lineages, which might reflect ecological differences within the species, notably estimates of dN/dS ratios in the *F. tularensis* subsp. *holarctica* clade were consistently higher than in the clade consisting of subspp. *tularensis* and *mediasiatica*.

**Figure 4:**
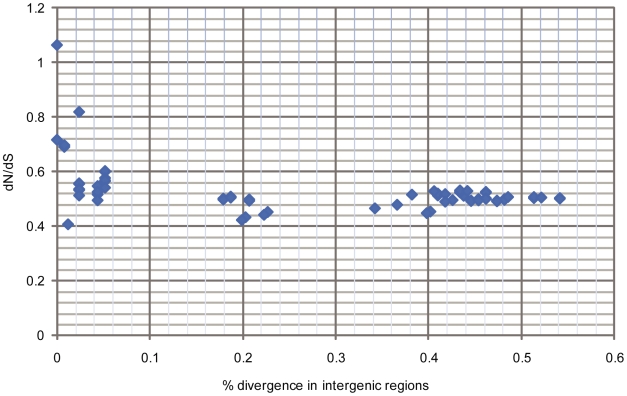
Illustrative plot of pairwise dN/dS values versus sequence divergence in intergenic regions among 13 *F. tularensis* strains. The high overall dN/dS values are indicative of inefficient purifying selection, and time-dependence at intergenic distances <0.15% is apparent.

It has been found that among closely related strains or species dN/dS ratios can be elevated in a time-dependent fashion [31],[32], and thus not reflect longer term selection pressures. This is because such comparisons are akin to the study of *de novo* mutations that have not yet been eliminated at the population level. Since all *F. tularensis* isolates are highly similar, we anticipated that there was a high likelihood that such effects would be observed. By plotting intergenic distances against dN/dS ratios determined in pairwise comparisons of 13 *F. tularensis* isolates, a negative correlation was indeed found between intergenic distance and dN/dS for the most closely related genomes, in support of non-stationarity (Figure 4). The correlation disappeared, however, at intergenic distances >0.15%, beyond which dN/dS ratios asymptotically approached a value of ∼0.5, suggesting that stationarity of dN/dS was reached. These findings indicate that, because of time dependence, dN/dS ratios will be inflated for branches between very closely related genomes (e.g. OSU18, FTA, LVS). The lineage-specific analyses of dN/dS ratios, however, were performed using less closely related genomes, a precaution taken to limit effects of time dependence. In analyses of genome-wide mutational biases, we noted a correspondence between a relative surplus of G+C→A+T mutations and a reduced level of purifying selection (Table S4 and Figure 3).

### Analysis of gene-inactivating mutations across multiple *Francisella* genomes

Among seven complete *Francisella* genome sequences (U112, FSC147, SCHUS4, WY-96, LVS, FTA, OSU18) there is an overall evolutionary pattern of step-by-step degradation of genes, which are ultimately deleted. The majority of gene disruptions found in modern *F. tularensis* strains occurred independently along the five major genetic branches of *F. tularensis* (Table S5, S6). That is, we see a converging evolutionary scenario among the *F. tularensis* lineages towards a common functional gene set. The strain *F. novicida* U112 has the least degraded, and largest, of all the analyzed genomes, containing 1,731 protein-coding genes and only 14 pseudogenes, according to recent annotation by Rhomer at al. [22]. A common set of 1,162 genes was identified that appeared to be functional in all seven genomes. This set of genes represents functions that have been preserved amongst *F. novicida* strain U112 and the six *F. tularensis* strains.

**Figure 5:**
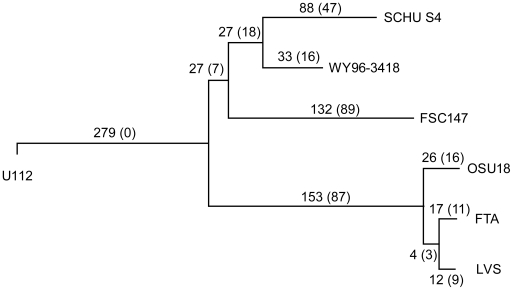
The distribution of gene function losses projected for the *Francisella* phylogeny. Total numbers of gene function losses are indicated on branches, and values in brackets indicate numbers of homoplastic gene function losses. Homoplastic loss means that these genes have been inactivated independently in different branches.

Next, we identified gene function losses across the reconstructed phylogeny using a parsimony criterion. The absence of a full-length gene in two terminal branches was taken to indicate an absence in the nodes connecting the branches. We identified 798 gene function losses, in total, across the phylogeny depicted in Figure 5, of which only ca. 62% (495/798) show inactivation patterns that are congruent with the inferred SNP phylogeny. In reality this is an overestimate of the proportion of congruent events, since in some cases there are likely to have been several independent disruptions of the same gene, which will remain undetected along internal branches including the branch from *F. novicida* to the last common ancestor of *F. tularensis* (279 congruent gene losses are indicated in Figure 5).We counted 166 gene losses and 109 predicted pseudogenes, in comparison with the *F. novicida* U112 genome. An additional four genes were either absent or are pseudogenes in the six *F. tularensis* strains, making the total of 279 gene function losses. The function of these genes was presumably lost after the divergence of *F. novicida* and *F. tularensis* from a common ancestor. An alternative scenario, of gene acquisition in the *F. novicida* branch, can easily be dismissed by inspecting intact and disrupted genes in the genomes, since we identified only 11 genes that are absent in *F. novicida* compared with the common *F. tularensis*-*F. novicida* gene set. Mapping of gene function losses on the SNP tree shows additional distinct losses along each of the major genetic branches of *F. tularensis*. The virtual absence of recombination among the *F. tularensis* lineages makes further analysis fruitful. It is clear that the majority of gene function losses have occurred independently along the branches (Figure 5).

We explored further by simulation possible characteristics of a putative dispensable gene set. A simplistic model was used with genes randomly sampled for inactivation under a uniform distribution model along branches according to frequencies previously identified by parsimony-mapping. Using this method, expected numbers of apparently homoplastic gene disruptions were inferred for different effective gene set sizes. The number of homoplasies in the real data was found to greatly exceed the numbers expected for any gene sample size, with a maximum of ∼80 homoplasies at an optimal gene sample size of 400 (Figure S1). An additional simulation was performed with increased numbers of homoplasies to account for the possibility that the true number of homoplasies may be greater than apparent numbers, but again similar results were obtained (data not shown).

### Analysis of IS element expansions and genomic rearrangements

**Figure 6:**
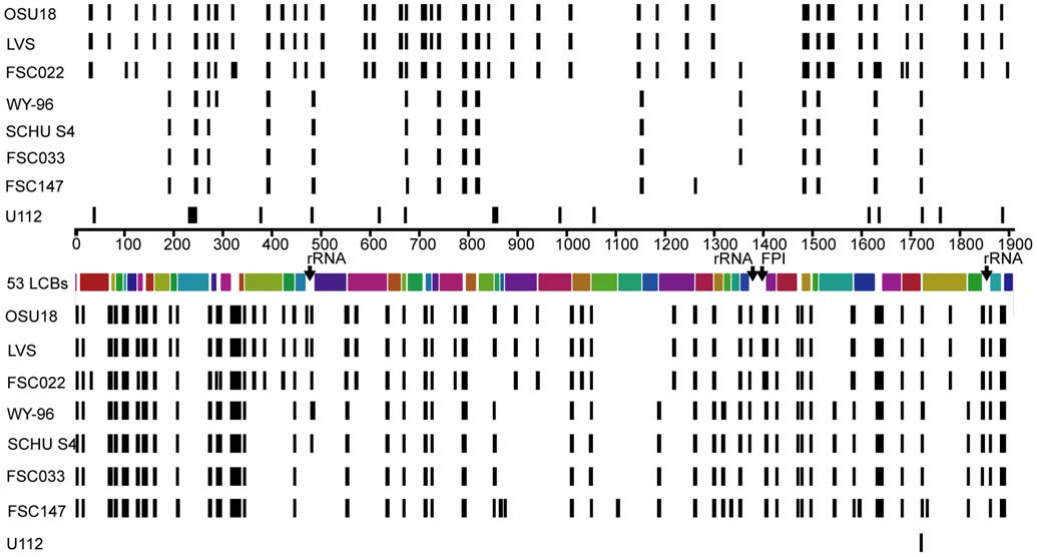
Gene order alterations in *F. tularensis* have mainly occurred with breakpoints at IS-elements. Results of BLASTN analyses of sequences flanking each ISFtu1 and ISFtu2 element in eight completed *Francisella* genomes, showing “hits” mapped along a linear depiction of the *F. novicida* U112 chromosome, with 53 local collinear sequence blocks within the 1,910 kb sequence. A black line in the upper panel corresponds to a flanking sequence of an ISFtu2 and a black line in the lower panel to a flank of an ISFtu1 element. The positions of rRNA genes and the FPI in the *F. novicida* U112 sequence are indicated.

In agreement with previous suggestions, we found strong indications that rearrangements in *F. tularensis* have been mediated by IS elements after divergence from a common *F. tularensis* ancestor [21],[22]. Mapping of flanking sequences of ISFtu1 and ISFtu2 in three completed *F. novicida* and eight *F. tularensis* genomes identified a most parsimonious scenario for the pattern and order in which the IS elements have been inserted during the course of evolution (Figure 6). The ISFtu2 distribution in the genomes was consistent with largely independent past increases in ISFtu2 numbers in *F. novicida* and *F. tularensis*. We counted 17 ISFtu2 elements in *F. novicida* U112, but found that only a single element had a corresponding flanking nucleotide sequence in a *F. tularensis* genome (at 246,100 bp in *F. novicida* U112). Thirteen ISFtu2 elements were likely inserted into ancestral *F. tularensis* taxa before the formation of the major genetic lineages of *F. tularensis*, since they share one or both flanking sequences in all *F. tularensis* genomes. The rate of expansion of ISFtu2 was reduced before the formation of the *tularensis*-*mediasiatica* genetic clade, seen as an extensive positional conservation of sequences flanking 16–18 ISFtu2 elements in both branches (Figure 6). During formation of the *holarctica* clade, ISFtu2 continued to be inserted at novel positions, resulting in a total of 42–44 occurrences in this subspecies. The ISFtu1 element appears to have a different evolutionary history. Mapping the flanking sequences of ISFtu1 shows that nearly all ISFtu1 elements found in *F. tularensis* strains (N = 44) were inserted before formation of subsp. *holarctica*, but later than the divergence of *F. novicida* (Figure 6). The patterns of IS elements also indicates that there were no individual expansions of ISFtu1 in the A1 or A2 clades of *F. tularensis* subsp. *tularensis*. No ISFtu1 border was unique comparing strain SCHU S4 with strain WY-96. Six unique ISFtu1 element insertions occurred along the *F. tularensis* subsp. *mediasiatica* lineage.

**Figure 7:**
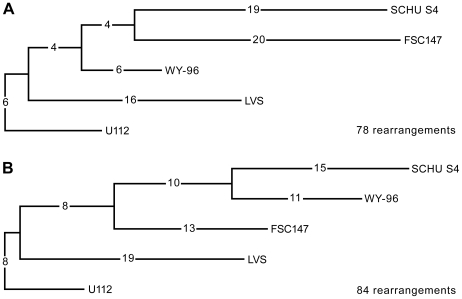
Phylogeny based on gene orders in six completed *F. tularensis* genomes (SCHU S4, WY-96, FSC147 and the identical orders of OSU18, LVS and FTA) and one *F. novicida* genome (U112). Inversion distances are indicated on the branches. Reconstructions were generated both without a constraining topology (A), and with the “true” topology, as determined by SNP analysis (B).

Using Multiple Genome Rearrangements, an inversion metric software package [33], to analyze the order of 53 local collinear sequence blocks (LCBs) in seven completed and aligned sequences, we observed large rearrangement distances despite close genetic relationships at the SNP level among strains (Figure 7). No less than 78 inversions were required to explain the gene order data. In reconstructions without imposing topological constraints, the algorithm was unable to recover the SNP-based tree. Assuming an ancestral gene order close to that of the species with the lowest number of IS elements (*F. novicida* U112) or a reconstructed order at its closest internal node, strain WY-96 displayed the lowest rearrangement distance (in accordance with the shortest SNP distance; Figure 7). The LVS lineage was inferred to have a shorter inversion distance than both SCHU S4 and FSC147. The gene orders of WY-96, SCHU S4 and FSC147 are highly divergent, resulting in large rearrangement distances despite their intimate relationships as determined by SNP analysis. Increased rates of rearrangements are therefore apparent in the *mediasiatica* FSC147 and *tularensis* A1 SCHU S4 lineages.

### Gene amplification in *F. tularensis* and the origin of the *Francisella* Pathogenicity Island

A set of 16–19 genes denoted the *Francisella* pathogenicity island (FPI) is critical for phagosomal escape, and is present in duplicate in *F. tularensis* strains, but in a single copy in the less virulent *F. novicida* [34],[35]. Given the importance of the FPI for intracellular replication, it is likely that the duplication represents an adaptation to a more restricted niche. Our analysis confirmed that the FPI exists in duplicate in all analyzed *F. tularensis* genomes (*F. tularensis* subspp. *holarctica*, *mediasiatica* and *tularensis*) and showed there were single copies of homologous genes in all the analyzed environmental *Francisella* genomes, i.e. *Francisella philomiragia* ATCC 25017, *F. novicida* U112, and the two *novicida*-like strains GA 99-3548 and GA 99-3549 (Figure S2). Its ubiquitous presence among strains of genus *Francisella* challenges the description of the FPI as a classic pathogenicity island, i.e., a mobile locus promoting pathogenicity with specific presence in pathogens but absence in benign relatives [36]. However, in agreement with its designation as a pathogenicity island we reaffirm that the region likely has a lateral origin and was inserted into an ancestor of *Francisella*. Further, in *Francisella*, FPI genes appear to be part of the core genome.

Analysis of proteins encoded within and outside the FPI demonstrated that the most over-represented amino acids within the FPI correspond to those encoded by the most GC-rich codon families, i.e. alanine, glycine, proline, arginine, tryptophan and cysteine. Accordingly, the most underrepresented amino acids are encoded by the most GC-poor codon families, i.e. isoleucine, tyrosine, aspargine, lycine and phenylalanine. Since the composition of encoded amino acids has been found to be strongly influenced by G+C content [37], this finding indicates that the FPI was originally acquired from an organism with a higher G+C content. In agreement with long presence of the FPI in *Francisella* we found no significant differences at third codon positions in G+C composition between the FPI and other parts of the genome (17% for non-ribosomal proteins encoded outside the FPI, and 16% for those encoded within the FPI).

**Figure 8:**
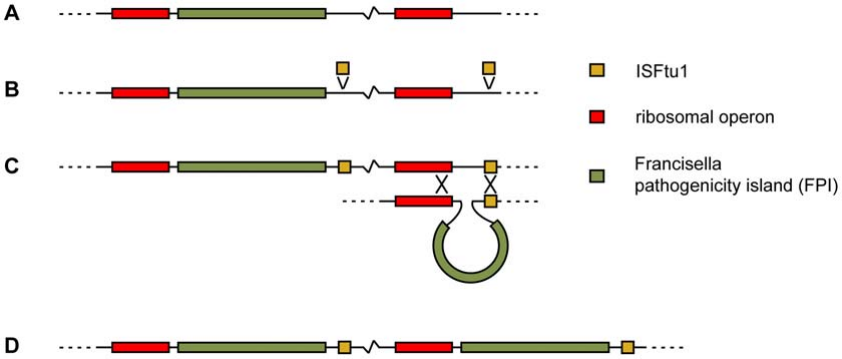
A proposed model for duplication of the FPI. (A) In an ancestral genome similar to *F. novicida* U112, the FPI was present as a single copy. (B) Insertion of copies of the ISFtu1 element adjacent to the present FPI, and adjacent to a second rRNA operon, enabled (C) duplication of the FPI by non-reciprocal recombination. (D) The two copies of the region have been inherited by all accepted *F. tularensis* subspecies.

The presence of an ISFtu1 insertion sequence element at one flank of the FPI has previously been assumed to have mediated its lateral transfer. However, irreconcilably with such a role, this ISFtu1 copy appears to have a more recent origin than the FPI itself, since it is present exclusively in members of *F. tularensis*. We infer that the flanking ISFtu1 instead likely played a role in the duplication of the FPI. As outlined in Figure 8, the most parsimonious evolutionary scenario appears to be that insertions of an ISFtu1 element adjacent to FPI genes and a set of rRNA genes occurred in a common *F. tularensis* ancestor, followed by duplication of FPI genes by unequal recombination. An ancestral FPI unit position can also be inferred from the observed conservation of surrounding genes in *F. novicida* U112, *F. tularensis* subsp. *tularensis* WY-96 and subsp. *holarctica* sequences. From its unique position among LCBs in different strains, it is also evident that the second copy has subsequently acted as an independent rearrangement unit in all *F. tularensis* lineages (data not shown).

The putative glycosyl transferase may represent a second example of gene multiplication in *Francisella* coinciding with a function that is central to the ecology of the bacterium. A gene which encodes a putative glycosyl transferase (FTT0354, FTT0378, FTT1263) is found in two to four copies in all *F. tularensis* genomes, but not among environmental *Francisella* genomes (*F. novicida* and *F. philomiragia*). This gene, unlike the FPI, may therefore have arisen in the *F. tularensis* lineage via a lateral gene transfer event. Again, flanking ISFtu1 elements seem to have subsequently mediated homologous recombination and gene multiplication events (not shown). Under the hypothesis that the gene acquisition and amplification reflect adaptive processes, we analyzed non-synonymous/synonymous mutation ratios, and found significant evidence of positive selection according to codeml estimates [38]. The M2a model indicated a dN/dS ratio of 2.344, and that 2.38% of the nucleotide sites have been positively selected. Likelihood ratio tests using both codeml model pairs M1a/M2a and M7/M8 indicated that the null hypothesis of neutral evolution should be rejected (*p* = 0.0242 and *p* = 0.0282, respectively).

## Discussion

We have performed a broad comparison of 17 *Francisella* genomes to infer past evolutionary events and possible ecological adaptations that distinguish the primarily human pathogenic *F. tularensis* from its less virulent opportunistic neighbors, *F. novicida* and *F. philomiragia*. Our analysis provides support for a proposed evolutionary scenario of events that formed *F. tularensis*. The analysis also offers important clarifications of several uncertainties and ambiguities regarding *F. tularensis*, more specifically concerning: (i) the phylogenetic origin of *F. tularensis* subsp *mediasiatica*, (ii) the suggested dependence of virulence on the mere occurrence of specific genes, (iii) the occurrence of genetic recombination in *F. tularensis*, and (iv) the previously suggested lateral mobility of the FPI in *F. tularensis*. In addition, the results provide evolutionary data indicating that strains of *F. novicida* should continue to be regarded as a separate species.

Based on our results, we propose the following sequence of events. *F. tularensis* emerged from a recombining *Francisella* population that was relatively unrestricted or free-living, then an ancestral *F. tularensis* variant invaded a novel and more host-restricted niche. This event led to clonal evolution under a reduced purifying selection pressure with ensuing genome degradation and proliferation of insertion sequence elements. We further propose that this series of events provided important prerequisites for further alterations of genomic architecture, and possibly increased adaptability of *F. tularensis*. In support of this scenario, we found apparent differences in the evolutionary mode of basal *Francisella* lineages (including *F. philomiragia*, *F. novicida* and an ancestral *F. tularensis* lineage), and *F. tularensis* lineages. These apparent differences include frequent genetic exchanges among basal *Francisella* lineages, but not among *F. tularensis* lineages, strong purifying selection pressure in basal lineages but weak levels in *F. tularensis*, and increasing frequencies of adenine and thymine nucleotides in *F. tularensis* but not in *F. novicida* genomes (Table S3). The pronounced increase in G+C→A+T mutations in *F. tularensis* supports a link to weak purifying selection, allowing for the fixation of slightly deleterious mutations. In agreement with this interpretation, Balbi et al. recently found inefficient purifying selection to be intimately connected with adenine and thymine enrichment in *Shigella* spp. [39].

Differences in genomic architecture were also apparent. While genome erosion appears to have occurred in all *F. tularensis* genomes, the representatives of basal lineages have maintained genomes tightly packed with genes. Corroborating a previous genomic analysis of *F. novicida* strain U112, the unfinished genomic sequences of *F. novicida* strain GA99-3548 and strain GA99-3549, as well as the completed *F. philomiragia* strain ATCC 25017, were found to contain few IS elements and pseudogenes. The overall gene synteny was extensive among the three available *F. novicida* genomes (data not shown). In contrast, all *F. tularensis* genomes are crowded with IS elements and pseudogenes, and display highly rearranged gene orders, each corresponding to a subspecies or a major genetic lineage. The findings in this study thus indicate that *F. novicida* has remained relatively unchanged over a long period with respect to gene content, presence of IS elements, and gene order. If so, the genomic architecture of the ancestor of *F. tularensis* must have more closely resembled *F. novicida* than any current *F. tularensis* isolate. Genomic data therefore indicate that the deviating evolutionary patterns in *F. tularensis* represent a derived state.

The greater metabolic competence of *F. novicida* compared to *F. tularensis*, and the abundance of IS elements in *F. tularensis* (but not *F. novicida*), provide additional indirect support for a change of living habitat. The genomic erosion identified in *F. tularensis* is consistent with its occupation of a habitat that supplies nutrients, making some metabolic functions superfluous. Host-pathogen or recent symbiotic restrictions appear to have been similarly associated with genome erosion and proliferation of IS elements in several other organisms, e.g. *Yersinia pestis* [40], *Bordetella pertussis* [41], and bacterial endosymbionts of insects [42]. Generally, IS element expansions in host-restricted bacteria are considered to be consequences of reductions in effective population size and relaxed purifying selection, which provide opportunities for insertions [3]. Supporting this hypothesis in *F. tularensis* is the bacterium's exceptionally high infectiousness, 10–25 cfu being sufficient to cause disease in humans, a trait consistent with repeated population contractions during infection of hosts.

Assuming that IS elements proliferate as a result of reduced selection pressure, it follows that this is a neutral process that in itself provides no advantage for the bacterium [43]. A neutral random insertion of IS elements likely provided the necessary raw materials for secondary pathoadaptive mutations in *F. tularensis*. Out of the two genetic loci that were found to be multiplied in all *F. tularensis* genomes by an IS element-mediated process, both were found to represent functions of central importance to the pathogen. The first locus corresponds to the FPI, a critical virulence determinant recognized for its importance for phagosomal escape [44]. The other locus contains a hypothetical glycosyltransferase gene, which we here demonstrate has been under strong adaptive selection. *F. tularensis* may therefore provide an example of an organism for which random genetic drift, with consequent fixation of many neutral or slightly deleterious mutations, provided novel evolutionary opportunities. Although not providing definitive proof, we propose that secondary gene multiplications enabled by past random IS element insertions represent examples of adaptively selected traits of the bacterium. In line with arguments recently advanced by Lynch [45], our data suggest that an accumulation of mutations that were originally neutral or slightly deleterious to the organism in the short term proved to be fruitful in the long term when exploited by natural selection.

As mentioned above, the data presented here also offer possible clarifications of several uncertain aspects and ambiguities regarding *F. tularensis*.

- One such ambiguity concerns the phylogenetic origin of *F. tularensis* subsp *mediasiatica*. We here identified *F. tularensis* subsp. *mediasiatica* strain FSC147 as a monophyletic *F. tularensis* taxon. This conflicts with observations by Nübel et al. [46], who found (using multi locus sequence typing) that *F. tularensis* subsp. *mediasiatica* strain FSC147 (denoted F68 in their study) is not a member of the *F. tularensis* clade, but instead is associated with environmental *Francisella* isolates. The finding that the subspecies *mediasiatica* is “phylogenetically incoherent” was a central conclusion in their work. However, we found that several gene fragment sequences for FSC147 deposited in GenBank by Nübel et al. differ from the genomic sequence of this strain, but coincide with *F. novicida* sequences. Thus, their conclusion of a polyphyletic origin of the subspecies *mediasiatica* requires re-appraisal.
- Another uncertainty concerns the suggested dependence of virulence on the mere presence of specific virulence genes [22]. Given the high genetic similarity between the members of subspp. *tularensis* and *mediasiatica*, there is an intriguing difference in virulence between the two subspecies [14]. In a recent genome comparison, Rhomer et al. suggested a set of nine genes to be candidate mediators of the high virulence of subsp. *tularensis* [22]. Our observations of gene content in the various subspecies of *F. tularensis*, including *F. tularensis* subsp. *mediasiatica*, provide little support for the hypothesis that any of these genes explain the higher degree of virulence of the *tularensis* subspecies. Our analyses indicate instead that these particular gene differences exemplify a superfluous gene set that is common to all *F. tularensis* lineages and is not yet completely inactivated in subspp. *tularensis* and *mediasiatica* (Figure 5 and Figure S1). Further, we found evidence for substantially reduced purifying selection in *F. tularensis*, implying that its evolution has been strongly affected by random genetic drift. These findings do not exclude pathoadaptation of individual lineages. Possibly, gene silencing in subsp. *tularensis* may have promoted virulence, as has been suggested for the host adaptation of *Shigella* and *Salmonella*, in which deletion mutants of specific genes have produced phenotypes of increased virulence [47],[48]. In *F. novicida*, there is a parallel example, since silencing the *pepO* gene promoted virulence in a mouse model [49]. Moreover, it is possible that virulence alterations may have resulted from genomic rearrangements during the formation of subspecies, affecting transcriptional networks.
- A third uncertainty regards the suggested occurrence of genetic recombination in *F. tularensis* [46]. Among lineages within *F. tularensis*, we found no evidence of past recombination events. Our recombination analyses suggest that the few homoplasies detected in *F. tularensis* instead likely arose as a consequence of mutational biases in *F. tularensis* (Table 3, Table S3). In contrast, our comparative genome sequence data show that recombination events have been common features of the evolution of all environmental lineages, here represented by *F. novicida* U112, *novicida*-like strains GA99-3548, GA99-3549 and the *F. philomiragia* strain ATCC 25017. Visual sequence analysis, inferred recombination rates by Clonalframe, and Hudson's Rm all indicate substantial recombination rates. Since Rm represents a lower bound and ClonalFrame only models recombination “imports”, both methods likely underestimate the true number of recombinations.
- A fourth uncertainty concerns the suggested lateral mobility of the FPI in *F. tularensis* [35]. We found that the FPI is ubiquitous across all investigated genomes, implying that it was incorporated at an early stage into a *Francisella* ancestor. It is likely that acquisition of the FPI genes (which are now permanently integrated in duplicate copies in the chromosome of all *F. tularensis* lineages) was an important event for early host adaptation of *Francisella*. We found no genetic traces of recent extra-chromosomal mobilization of the FPI in *F. tularensis*, instead these genes seem to have evolved into a duplicated part of the core genome.

**Table 3:** Results of test for recombination among 13 *F. tularensis* genomes.

	Sites at risk, *N*	Effective sites/sites at risk, *S*_e_/*S*	Homoplasies	Homoplasies expected if clonal, *h*_c_ (range)
Values inferred from real data	566,154	0.369	21	
Simulations at different *S*_e_/*S*	566,154	0.37		32.3 (23–42)
	566,154	0.50		24.1 (16–32)
	566,154	0.70		16.7 (10–24)

Finally, the results provide compelling arguments in favour of continuing to regard strains of *F. novicida* as belonging to a separate species. In agreement with a previous proposal by Hollis et al., based on DNA-DNA re-association [50], our ANI analysis indicates that strains belonging to *F. novicida* meet formal requirements for classification as a *F. tularensis* subspecies. All pairs of isolates classified as *F. novicida*, *novicida*-like, and *F. tularensis* demonstrated ANI values well above 95% (Table S1), a limit proposed as the threshold for classification into different bacterial species [51]. According to the method-free species concept recently outlined by Wagner and Achtman [52], however, species should be regarded as “metapopulation lineages” where separate designations are warranted if population lineages evolved separately despite a close relatedness. Our comparisons of environmental lineages (*F. novicida*, *F. philomiragia*) and *F. tularensis* show a typical example of such evolutionary separation. In addition to distinct population structures with regard to recombination, we also found substantial differences in overall dN/dS between environmental *Francisella* and *F. tularensis* (Figure 3), lending support to smaller effective population sizes in the latter. Other differences between environmental *Francisella* and *F. tularensis* include differences in metabolic competence, which is higher among environmental strains, and signs of ongoing genome erosion, which is pronounced among *F. tularensis* strains but not among the analyzed *F. philomiragia* and *F. novicida* strains.

It is also clear that tularemia caused by *F. tularensis* is a distinct clinical disease entity with little similarity to the bacteraemia caused by *F. novicida* [50],[53]. Moreover, tularemia is a classical vector-borne zoonosis while *F. novicida* is not known to be transmitted among vertebrate species, and *F. tularensis* is considered a biothreat agent while *F. novicida* is not. A fuzzy distinction between these quite different organisms may therefore complicate clinical decisions. Based on the evolutionary analyses described in this work, their distinct epidemiological features, and on clinical grounds: even though their average nucleotide identities exceed 97%, we propose that the species boundary between *F. tularensis* and *F. novicida* should be retained.

## Materials and Methods

### Genome sequencing

DNA for genomic sequencing of *F. tularensis* subsp. *mediasiatica* FSC147 was prepared as described in Text S1. The genome was sequenced at the Joint Genome Institute using small (2–3 kb) and medium (6–8 kb) insert plasmid libraries. The Phred/Phrap/Consed software package was used for sequence assembly and quality assessment [54]. During the manual finishing process, possible mis-assemblies were corrected by transposon bombing (Epicentre Biotechnologies) of bridging clones. Gaps between contigs were closed by editing in Consed, by custom primer walks, or by PCR amplification.

### Genome annotation

The nucleic acid sequence and annotation of *F. tularensis* subsp. *mediasiatica* strain FSC147 was deposited in GenBank under accession no. CP000915.1. Automatic annotation using TIGR's annotation engine for gene prediction, GO classification, EC numbers, and protein functions was performed. The annotation was then manually curated with the aid of previous annotations of *F. novicida* strain U112 and *F. tularensis* subsp. *tularensis* strain SCHUS4. Information on these and other genomes used in this study is presented in Table S1.

### Multiple alignments

All multiple alignments of genomic sequences were performed using Mauve v. 2.2 [55] with the progressive alignment option under default parameters. All alignments were visually inspected and potentially incorrectly aligned regions were removed before further analysis.

### Phylogeny

Phylogenetic analyses were conducted using MEGA version 4 [56] and Phyml v.2.4.4 [57]. Using 1,104,129 aligned sites from all 17 taxa (Table S1), Mega was used for Neighbor-joining based estimates of the *Francisella* phylogeny and 1,000 bootstrap pseudo-replicates were performed. The evolutionary distances were computed using Tamura's three-parameter distance model. For maximum likelihood-based analysis, a subset of 566,154 randomly selected aligned nucleotide sites were used to avoid software crashes using the Phyml package. The GTR model with a proportion of invariant sites and six gamma-distributed discrete rate categories was used, estimated from the data. Non-parametric bootstrapping was performed using 100 pseudo-replicates in the maximum likelihood analysis.

### Estimation of evolutionary distances

Average nucleotide identity (ANI) estimates were obtained by whole-genome sequence comparisons. Using the Perl script language and the NCBI blastall package v. 2.2.17, we implemented the algorithm and performed analyses as previously described elsewhere [51].

### Analysis of recombination

Analysis of recombination in basal parts of the phylogeny was assessed using the ClonalFrame algorithm [25]. Default parameters were used except that the topology was fixed to that estimated from phylogenetic analyses to increase the computational speed. Mauve-alignments for the analysis were based on genomic sequences of the strains GA99-3548, GA99-3549, *F. novicida* U112, and *F. tularensis* LVS (Table S1). After manual curation to remove poorly aligned regions, 1,527,362 nucleic acid sites in 128 local collinear blocks were retained for the analysis. Markov chain Monte Carlo iterations were run for 500,000 generations

The proportion of genes affected by recombination was assessed by the MaxChi2 method [27] and the Phi method, both implemented in the Phi Package [28] (**p<0.01).

Recombination among *F. tularensis* lineages was analyzed using the homoplasy test as proposed by Maynard Smith and Smith [29], implementing the algorithm as a Perl script (available upon request) on genomic information from 13 *F. tularensis* isolates (Table S1). The effective number of mutable sites (*S_e_*), required by the method, was calculated as previously described [58]:
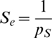
where *p_S_* is the probability that two independent substitutions in the gene occur at the same site. Since *F. tularensis* lineages deviate significantly from mutational equilibrium, we avoided using the “outgroup method” proposed by Maynard Smith and Smith [29]. This approach would have overestimated *S_e_* in our case because of the strong deviation from stationary nucleotide frequencies observed in *F. tularensis* (Table S3). Instead, we calculated *p_S_* using the number of sites (*nA*, *nC*, *nG*, *nT*), by their probability of mutation (*p_A_*, *p_C_*, *p_G_*, *p_T_*) as follows.

Given that two independent mutations occur. For all sites (*i*, *j*, *k*, *l*):
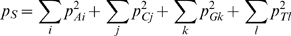
Where
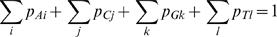
Estimates of numbers of sites and their probability of mutation were obtained from inferred ancestral nucleotide frequencies and mutations along the *F. tularensis* LVS branch from its division from the *F. tularensis* subsp. *tularensis* lineage, represented by the subsp. *tularensis* SCHU S4 strain. The reconstruction was performed using a maximum parsimony method in which only two-fold degenerate sites were used and the genomic sequence of *F. novicida* U112 was included as an outgroup. Sites with variable amino acids, changes at the first site of a codon, and sites coding for tryptophan and methionine residues were excluded from analysis to reduce the impact of selection.

Analysis of recombination was also performed by visual examination along genomic alignments of segregating sites, parsimony-informative sites and homoplasies. Genome-wide plots to support the analysis were generated using in-house Perl scripts.

### Analyses of selection

Assessment of positive selection for the multiplicated glycosyl transferase gene (corresponding to locus tags FTT0354, FTT0378, and FTT1263 in the *F. tularensis* SCHU S4 genomic annotation) was performed using Codeml in the Paml 4b package [38]. The probability of positive selection was assessed by likelihood ratio tests (LRT) for the hierarchical model pairs M1a vs. M2a and M7 vs. M8.

The HYPHY package [30] was used to assess branch-specific selectional regimes in *Francisella*. Two sets of analyses were performed. In one, entirely local models were fitted to the data and estimates of dN and dS were allowed to vary freely for each branch. Confidence intervals were here determined using the asymptotic normality of the maximum likelihood estimates. In the other, branch estimates of dN/dS were obtained using a genetic algorithm [30], ensuring that the data had not been overfitted. In all analyses, the Muse-Gaut 94 (MG94) 3×4 model [59] crossed with the general time-reversible (GTR) model was used, justified by parametric bootstrapping (in comparison with the Goldman-Yang 94, GY94, model [60]) and by likelihood ratio tests.

For analysis of time-dependence of dN/dS estimates, pairwise estimates of synonymous, non-synonymous, and intergenic evolutionary distances between isolates was estimated using MEGA version 4 [56] using the Nei-Gojobori method [61].

### Detection of pseudogenes and analysis of gene-inactivating mutations

A modified version of the Psi-Phi package [62] was applied using U112 as the reference genome to identify pseudogenes. The default settings for Psi-Phi were used except for the merging distance, which was set to 1350 to allow for ISFtu1 insertion events. A parsimony criterion was applied to determine the functional status of genes in internal nodes of a whole genome SNP phylogeny. The absence of a full length gene in two terminal branches was taken to indicate an absence in the nodes connecting the branches. The sum of gene deletions and pseudogenes constituted the total amount of gene function loss. Both congruent and homoplastic gene function losses were considered, using the whole genome SNP tree as a reference.

### Inference of genome level rearrangements

An alignment including 1,762,117 nucleotides in *F. novicida* U112 was used to explore genome-level rearrangements. Duplicated sequences including ribosomal RNA genes, the 30–34 kb duplicated sequence, and all IS elements were masked prior to rearrangement analysis. The LCB-weight was set to remove any very short collinear blocks, since we reasoned that these LCBs may be prone to duplication followed by random deletion events, which could lead to incorrect reconstructions. For calculating rearrangement scenarios based on inversions, the five gene orders representing strains U112, SCHU S4, WY-96, and the common gene orders for LVS, FTA and OSU18 were analyzed using MGR software [33], run in the circular genomes mode both with and without a fixed tree topology.

### Accession numbers

Locus tags referred to in the text correspond to those used in the annotation of the *F. tularensis* SCHU S4 genomic sequence (AJ749949): FTT0354, FTT0378, FTT1066-FTT1073, FTT1263, FTT1308c, FTT1580c, FTT1581-FTT1582 and FTT1791.

Completed genomic sequences with accession numbers used in this work are: U112 (CP000439), ATCC 25017 (CP000937), WY96-3418(CP000608), FSC147 (CP000915), FTA/FTNF002-00 (CP000803), OSU18 (CP000437), LVS (AM233362), SCHU S4 (AJ749949).

Preliminary sequence data were obtained from the MIT Broad Institute website at www.broad.mit.edu for the following *Francisella* strains, GA99-3549, GA99-3548, FSC033, FSC022, and FSC257, and from the Baylor College of Medicine Human Genome Sequencing Center website at www.hgsc.bcm.tmc.edu for the following *Francisella* strains: ATCC 6223, KO 97-1026, MI 00-1730 and OR 96-0246.

## Supporting Information

Figure S1 Monte Carlo simulation of numbers of homoplastic gene function losses that can be expected from random sampling of genes in completed *F. tularensis* genomes. The simulation suggests an upper limit of 80 homoplastic events to occur via stochastic mechanisms in a set of 400 sampled genes. Higher or lower numbers of genes result in lower numbers of homoplastic events.
(0.57 MB TIF)

Figure S2 The genes of the FPI are ubiquitous in the analyzed *Francisella* genomes and cannot have been recently introduced into the genus. Open reading frames and their orientation in different genomes are indicated by arrows. Gene orders are given with *F. novicida* (Fn), *F. tularensis* subsp. *tularensis* (Ftt) and *F. tularensis* subsp *mediasiatica* (Ftm) as references. It can be seen that genomic organization is similar in *F. tularensis* subsp. *holarctica* (Fth) and *F. philomiragia* (Fp). For completeness we also show a second region of Fn U112 that has partial homology with the gene cluster that has been denoted the FPI.
(0.30 MB TIF)

Table S1 Seventeen *Francisella* genomes that were analyzed in this study.
(0.05 MB DOC)

Table S2 Presence and absence of putative DNA repair enzymes in different *Francisella* isolates.
(0.09 MB DOC)

Table S3 Cross-table with percent pair wise average nucleotide identity values for 17 *Francisella* genomes.
(0.08 MB DOC)

Table S4 Single nucleotide mutations along terminal branches of *Francisella* taxa as inferred according to a parsimony criterion.
(0.04 MB DOC)

Table S5 Presence and absence of pseudogenes in different *F. tularensis* genomes as determined by Psi-Fi with *F. novicda* U112 (acc. CP000439) as reference.
(0.77 MB DOC)

Table S6 Missing genes in different *F. tularensis* genomes as compared to *F. novicida* U112 (GenBank acc. no. CP000439). Zero denotes the absence and one the presence of a gene.
(0.31 MB DOC)

Text S1 Preparation of genomic DNA.
(0.03 MB DOC)
