# Supplementary figures and images for: Molecular Evolutionary Consequences of Niche Restriction in Francisella tularensis, a Facultative Intracellular Pathogen
Source: PLoS Pathog. 2009 Jun 12;5(6):e1000472. doi: 10.1371/journal.ppat.1000472 (PMC2688086; doi:10.1371/journal.ppat.1000472)

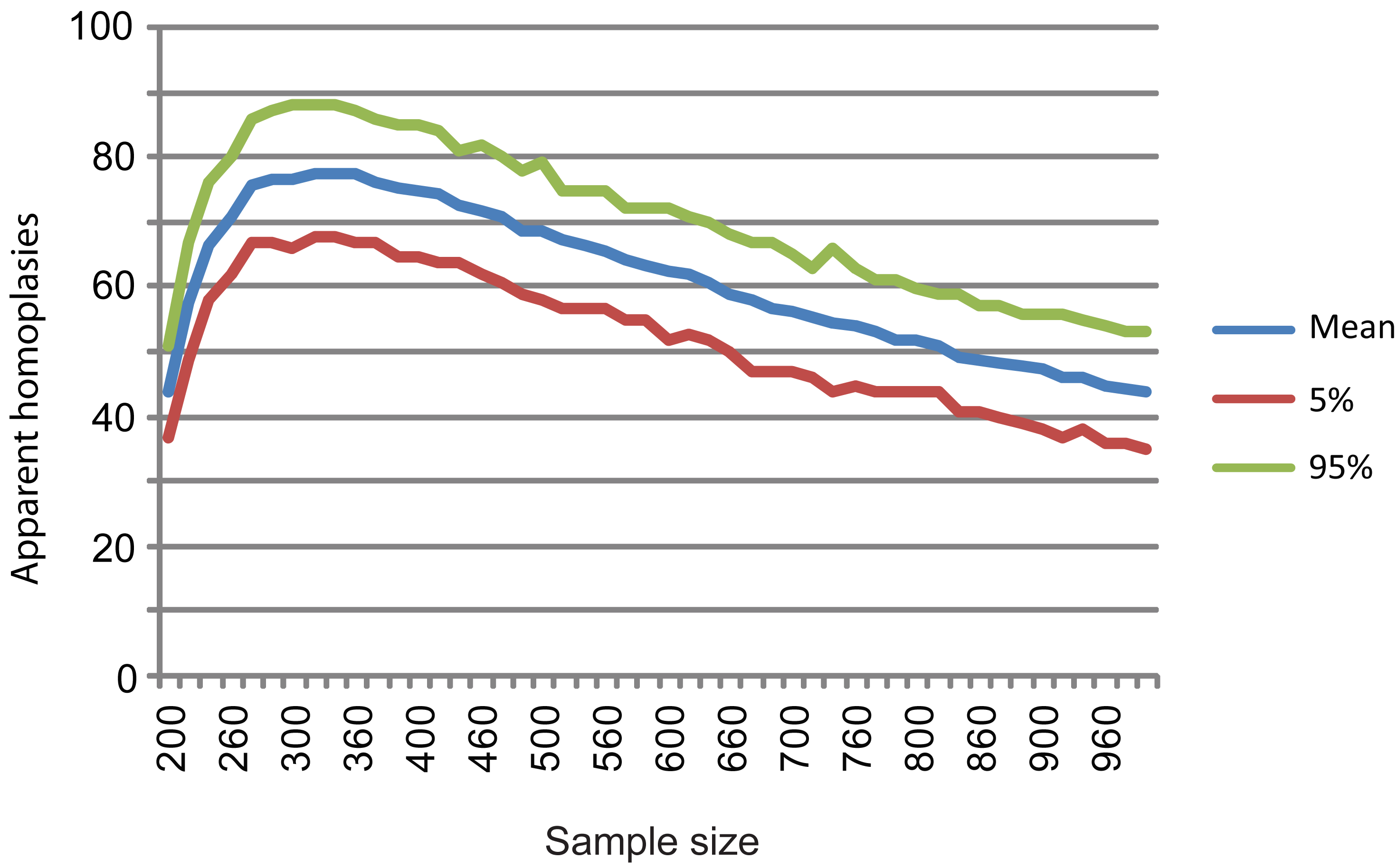

Supplement: Figure S1 — Monte Carlo simulation of numbers of homoplastic gene function losses that can be expected from random sampling of genes in completed F. tularensis genomes. The simulation suggests an upper limit of 80 homoplastic events to occur via stochastic mechanisms in a set of 400 sampled genes. Higher or lower numbers of genes result in lower numbers of homoplastic events. (0.57 MB TIF) [file ppat.1000472.s001.tif]

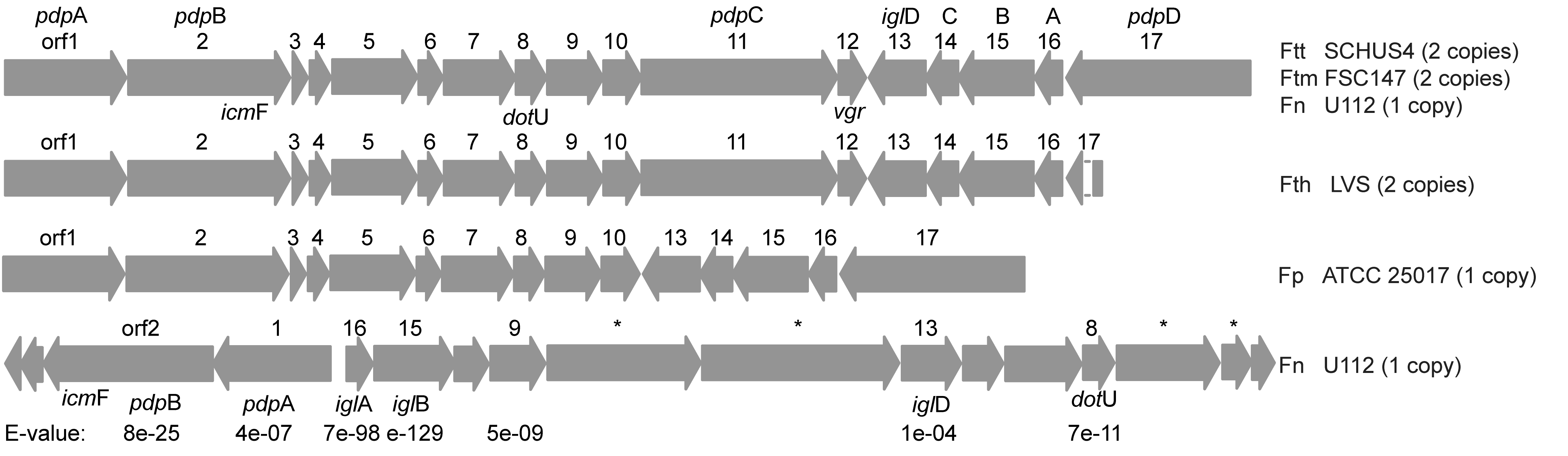

Supplement: Figure S2 — The genes of the FPI are ubiquitous in the analyzed Francisella genomes and cannot have been recently introduced into the genus. Open reading frames and their orientation in different genomes are indicated by arrows. Gene orders are given with F. novicida (Fn), F. tularensis subsp. tularensis (Ftt) and F. tularensis subsp mediasiatica (Ftm) as references. It can be seen that genomic organization is similar in F. tularensis subsp. holarctica (Fth) and F. philomiragia (Fp). For completeness we also show a second region of Fn U112 that has partial homology with the gene cluster that has been denoted the FPI. (0.30 MB TIF) [file ppat.1000472.s002.tif]
