# Supplementary material for: Molecular Evolutionary Consequences of Niche Restriction in Francisella tularensis, a Facultative Intracellular Pathogen
Source: PLoS Pathog. 2009 Jun 12;5(6):e1000472. doi: 10.1371/journal.ppat.1000472 (PMC2688086; doi:10.1371/journal.ppat.1000472)
Supplement: Table S1 — Seventeen Francisella genomes that were analyzed in this study. (0.05 MB DOC) [file ppat.1000472.s003.doc]

Table S1. Seventeen *Francisella* genomes that were analyzed in this study

| Species and subspecies name (genetic group) | Strain name | FSC^a^ no. | ATCC^b^ no. | Place of isolation | Source of genomic sequence | Status | GenBank  accession number |
| --- | --- | --- | --- | --- | --- | --- | --- |
| *F. novicida* | U112 | - | 15482 | Utah | University of Washington, WA | completed | CP000439 |
| “F. novicida-like” | F6168/GA 99-3549 | - | - | California | Broad Institute, MA | unfinished |  |
| “F. novicida-like” | D9876/GA 99-3548 | - | - | Louisiana | Broad Institute, MA | unfinished |  |
| *F. philomiragia* |  | - | 25017 | Utah | Lawrence Livermore National Laboratory, CA | unfinished |  |
| *F. tularensis* subsp. *tularensis* (A1) | SCHU S4 | 237 | - | Ohio | Swedish/British/US consortium^d^ | completed | AJ749949 |
| *F. tularensis* subsp. tularensis (A1) | SnMF | 033 | - | United States | Broad Institute, MA | unfinished | - |
| *F. tularensis* subsp. *tularensis* (A2) | WY96-3418 | - | - | Wyoming | Joint Genome Institute (JGI), Los Alamos National Laboratory, NM | completed | CP000608 |
| *F. tularensis* subsp. *tularensis* (A2) | B-38 | 230 | 6223 | Utah | Baylor College of Medicine, TX | unfinished | - |
| *F. tularensis* subsp. *mediasiatica* | GIEM 543 | 147 | - | Kazakstan | JGI, Los Alamos National Laboratory, NM | completed | CP000915 |
| *F. tularensis* subsp. *holarctica* | Ebina | 022 | - | Japan | Broad Institute, MA | unfinished | - |
| *F. tularensis* subsp. *holarctica* | GIEM 503 | 257 | - | Russia | Broad Institute, MA | unfinished |  |
| *F. tularensis* subsp. *holarctica* | KO 97-1026 | - | - | Korea | Baylor College of Medicine, TX | unfinished |  |
| *F. tularensis* subsp. *holarctica* | FTA/ FTNF002-00 | - | - | France | Joint Genome Institute (JGI), Los Alamos National Laboratory, Los Alamos, NM | completed |  |
| *F. tularensis* subsp. *holarctica* | MI 00-1730 | - | - | Michigan | Baylor College of Medicine, TX | unfinished |  |
| *F. tularensis* subsp. *holarctica* | OR 96-0246 | - | - | Oregon | Baylor College of Medicine, TX | unfinished |  |
| *F. tularensis* subsp. *holarctica* | OSU18 | - | - | Oklahoma | Baylor College of Medicine, TX | completed | CP000437 |
| *F. tularensis* subsp. *holarctica* | LVS |  | 29684 | Russia | Lawrence Livermore National Laboratory, CA | completed | AM233362 |

^a^*Francisella* Strain Collection. ^b^American Type Culture Collection.
