# Supplementary material for: Molecular Evolutionary Consequences of Niche Restriction in Francisella tularensis, a Facultative Intracellular Pathogen
Source: PLoS Pathog. 2009 Jun 12;5(6):e1000472. doi: 10.1371/journal.ppat.1000472 (PMC2688086; doi:10.1371/journal.ppat.1000472)
Supplement: Table S2 — Presence and absence of putative DNA repair enzymes in different Francisella isolates. (0.09 MB DOC) [file ppat.1000472.s004.doc]

| Putative protein function | Ref. Genbank accession | Gene | SCHU S4^a^ | WY-96^b^ | FSC 147^c^ | FSC 022^d^ | LVS^d^ | OSU 18^d^ | FTA^g^ | GA99- 3548^e^ | GA99- 3549^e^ | U112^f^ | ATCC 25017^g^ |
| --- | --- | --- | --- | --- | --- | --- | --- | --- | --- | --- | --- | --- | --- |
| transcription-repair coupling factor | A0Q6R1 | Mdf | + | + | + | + | + | + | + | + | + | + | + |
| DNA mismatch repair protein | A0Q5F7 | MutL | + | + | + | + | + | + | + | + | + | + | + |
| formamidopyrimidine-DNA glycosylase | A0Q5I3 | MutM | + | + | + | + | + | + | + | + | + | + | + |
| DNA mismatch repair protein | A4IXL2 | MUTS | + | + | + | + | + | + | + | + | + | + | + |
| endonuclease III | A0Q6Q7 | Nth | + | + | + | + | + | + | + | + | + | + | + |
| deoxyribodipyrimidine photolyase | A0Q6Z2 | PhrB | - | + | - | - | - | - | - | + | + | + | + |
| DNA polymerase I | A0Q899 | PolA | + | + | + | + | + | + | + | + | + | + | + |
| DNA repair protein radA | A0Q4Y7 | RadA | + | + | + | + | + | + | + | + | + | + | + |
| recombinase A protein | A0Q468 | RecA | + | + | + | + | + | + | + | + | + | + | + |
| Exodeoxyribonuclease V beta chain | A0Q7L7 | RecB | + | + | + | + | + | + | + | + | + | + | + |
| DNA replication and repair protein | A0Q5W0 | RecF | + | + | + | + | + | + | + | + | + | + | + |
| single-stranded-DNA-specific exonuclease | A0Q5E9 | RecJ | + | + | + | + | + | + | + | + | + | + | + |
| DNA repair protein N | A0Q500 | RecN | + | + | + | + | + | + | + | - | + | + | + |
| DNA repair protein | A0Q7A9 | RecO | + | + | + | + | + | + | + | + | + | + | + |
| recombination protein RecR | A0Q768 | RECR | + | + | + | + | + | + | + | + | + | + | + |
| holliday junction DNA helicase B | A0Q6B4 | RUVB | + | + | + | + | + | + | + | + | + | + | + |
| holliday junction endodeoxyribonuclease | A0Q6P9 | RUVC | + | + | + | + | + | + | + | + | + | + | + |
| resolvase/RNaseH-like | A0Q690 | RUVX | + | + | + | + | + | + | + | + | + | + | + |
| 3-methyladenine DNA glycosylase | A0Q5Q0 | tag | - | - | - | - | - | - | - | + | + | + | + |
| DNA polymerase IV | A0Q6K9 | UmuC | - | - | + | + | + | + | + | + | + | + | + |
| uracil-DNA glycosylase | A0Q7Y8 | UNG | + | + | + | + | + | + | + | + | + | + | + |
| DNA excision repair enzyme, subunit A | A0Q5P3 | UVRA | + | + | + | + | + | + | + | - | + | + | + |
| excinuclease ABC subunit B | Q5NFN4 | UVRB | + | + | + | + | + | + | + | + | + | + | + |
| excinuclease ABC subunit C | A0Q5Q7 | UvrC | + | + | + | + | + | + | + | + | + | + | + |
| excinuclease ABC subunit C | A0Q506 | UvrC | + | + | + | + | + | + | + | - | + | + | + |
| excinuclease ABC subunit C | A4IWI5 | UvrC | + | + | + | + | + | + | + | - | + | + | + |
| ATP-dependent DNA helicase | A0Q6+2 | UvrD | + | + | + | + | + | + | + | + | + | + | + |
| ATP-dependent DNA helicase | A0Q889 | UvrD | + | + | + | + | + | + | + | + | + | + | + |
| Met DNA-protein cysteine methyltransferase | A4IXJ6 |  | + | + | + | + | + | + | + | + | + | + | + |
| deoxyribodipyrimidine photolyase-rel. protein | A0Q4V3 |  | - | - | - | + | + | + | + | + | + | + | + |
| DNA ligase, NAD-dependent | A4KQL9 |  | + | + | + | + | + | + | + | + | + | + | + |
| methylpurine-DNA glycosylase family protein | A7JBU+ |  | + | + | - | + | + | + | + | + | + | - | - |
| deoxyribodipyrimidine photolyase | A7JG82 |  | + | + | + | - | - | - | - | + | + | + | + |

Table S2. Presence and absence of putative DNA repair enzymes in different *Francisella* isolates.

^a^ *F. tularensis* subsp. *tularensis* A1, ^b^ *F. tularensis* subsp. *tularensis* A2, ^c^ *F. tularensis* subsp. *mediasiatica*, ^d^ *F. tularensis* subsp. *holarctica*, ^e^ *F. novicida*-like bacterium, ^f^*F. novicida*, ^g^*F. philomiragia*.
