# Supplementary material for: Molecular Evolutionary Consequences of Niche Restriction in Francisella tularensis, a Facultative Intracellular Pathogen
Source: PLoS Pathog. 2009 Jun 12;5(6):e1000472. doi: 10.1371/journal.ppat.1000472 (PMC2688086; doi:10.1371/journal.ppat.1000472)
Supplement: Table S3 — Cross-table with percent pair wise average nucleotide identity values for 17 Francisella genomes. (0.08 MB DOC) [file ppat.1000472.s005.doc]

Table S3. Cross-table with percent pair wise average nucleotide identity values for 17 *Francisella* genomes.

|  |  |  |  |  |  |  |  |  |  |  |  |  |  |  |  |  |  |
| --- | --- | --- | --- | --- | --- | --- | --- | --- | --- | --- | --- | --- | --- | --- | --- | --- | --- |
|  |  | ATCC 25017 | U112 | GA99-3549 | GA99-3548 | SCHU S4 | FSC 033 | WY96-3418 | ATCC 6223 | FSC 147 | FSC 022 | OSU 18 | MI00-1730 | LVS | RC503 | OR96-0246 | FTA |
| *F. philomiragia* | ATCC 25017 |  |  |  |  |  |  |  |  |  |  |  |  |  |  |  |  |
| *F. novicida* | U112 | 81.26 |  |  |  |  |  |  |  |  |  |  |  |  |  |  |  |
|  | GA99-3549 | 80.87 | 98.40 |  |  |  |  |  |  |  |  |  |  |  |  |  |  |
|  | GA99-3548 | 80.95 | 98.53 | 98.19 |  |  |  |  |  |  |  |  |  |  |  |  |  |
| *F. t. t*.^a^ (A1) | SCHU S4 | 80.88 | 98.20 | 97.78 | 98.12 |  |  |  |  |  |  |  |  |  |  |  |  |
|  | FSC033 | 80.75 | 98.18 | 97.80 | 98.12 | 99.96 |  |  |  |  |  |  |  |  |  |  |  |
| *F. t. t*. (A2) | WY96-3418 | 80.93 | 98.26 | 97.86 | 98.21 | 99.69 | 99.66 |  |  |  |  |  |  |  |  |  |  |
|  | ATCC 6223 | 80.80 | 98.30 | 97.87 | 98.23 | 99.71 | 99.70 | 99.97 |  |  |  |  |  |  |  |  |  |
| *F. t. m.*^b^ | FSC147 | 80.85 | 98.13 | 97.73 | 98.08 | 99.46 | 99.44 | 99.53 | 99.57 |  |  |  |  |  |  |  |  |
| *F. t. h.*^c^ | FSC022 | 80.87 | 98.04 | 97.74 | 98.06 | 99.38 | 99.37 | 99.45 | 99.52 | 99.31 |  |  |  |  |  |  |  |
|  | OSU18 | 80.92 | 98.08 | 97.74 | 98.02 | 99.34 | 99.31 | 99.40 | 99.47 | 99.26 | 99.56 |  |  |  |  |  |  |
|  | MI00-1730 | 80.77 | 98.05 | 97.76 | 98.02 | 99.41 | 99.39 | 99.47 | 99.49 | 99.34 | 99.63 | 99.98 |  |  |  |  |  |
|  | LVS | 80.86 | 98.09 | 97.74 | 98.04 | 99.36 | 99.34 | 99.43 | 99.48 | 99.29 | 99.60 | 99.90 | 99.92 |  |  |  |  |
|  | RC503 | 80.78 | 98.05 | 97.75 | 98.03 | 99.41 | 99.40 | 99.48 | 99.50 | 99.34 | 99.68 | 99.93 | 99.98 | 99.98 |  |  |  |
|  | OR96-0246 | 80.78 | 98.06 | 97.77 | 98.04 | 99.41 | 99.40 | 99.48 | 99.50 | 99.34 | 99.67 | 99.92 | 100.0 | 99.92 | 99.90 |  |  |
|  | FTA | 80.90 | 98.07 | 97.75 | 98.03 | 99.36 | 99.35 | 99.42 | 99.48 | 99.28 | 99.60 | 99.90 | 99.93 | 99.91 | 99.93 | 99.97 |  |
|  | KO97-1026 | 80.78 | 98.05 | 97.78 | 98.04 | 99.40 | 99.39 | 99.48 | 99.50 | 99.34 | 99.65 | 99.92 | 99.96 | 99.92 | 99.95 | 99.88 | 100.0 |
|  |  |  |  |  |  |  |  |  |  |  |  |  |  |  |  |  |  |

^a^ *F. t. t.*: *F. tularensis* subsp. *tularensis*. ^b^ *F. t. m.*: *F. tularensis* subsp. *mediasiatica*. ^c^ *F. t. h.*: *Francisella tularensis* subsp. *holarctica*.
