# Supplementary material for: Molecular Evolutionary Consequences of Niche Restriction in Francisella tularensis, a Facultative Intracellular Pathogen
Source: PLoS Pathog. 2009 Jun 12;5(6):e1000472. doi: 10.1371/journal.ppat.1000472 (PMC2688086; doi:10.1371/journal.ppat.1000472)
Supplement: Table S4 — Single nucleotide mutations along terminal branches of Francisella taxa as inferred according to a parsimony criterion. (0.04 MB DOC) [file ppat.1000472.s006.doc]

Table S4. Single nucleotide mutations along terminal branches of *Francisella* taxa as inferred according to a parsimony criterion.

| Mutation | SCHU S4^a^ | FSC147^b^ | LVS^c^ | GA99-3549^d^ | U112^e^ | GA99-3548^f^ |
| --- | --- | --- | --- | --- | --- | --- |
| A→C | 3 | 6 | 9 | 70 | 69 | 67 |
| A→G | 73 | 157 | 69 | 661 | 541 | 723 |
| A→T | 14 | 13 | 8 | 223 | 156 | 193 |
| C→A | 29 | 23 | 37 | 99 | 64 | 99 |
| C→G | 2 | 2 | 0 | 16 | 21 | 23 |
| C→T | 130 | 192 | 298 | 749 | 563 | 707 |
| G→A | 151 | 140 | 225 | 576 | 478 | 546 |
| G→C | 1 | 0 | 0 | 23 | 17 | 22 |
| G→T | 15 | 13 | 26 | 90 | 54 | 114 |
| T→A | 14 | 24 | 21 | 260 | 175 | 244 |
| T→C | 85 | 131 | 74 | 727 | 643 | 772 |
| T→G | 10 | 19 | 15 | 94 | 75 | 92 |
| GC→AT | 325 | 368 | 586 | 1514 | 1159 | 1466 |
| AT→GC | 171 | 313 | 167 | 1552 | 1328 | 1654 |
| (GC→AT)/(AT→GC) | 1.90 | 1.18 | 3.51 | 0.98 | 0.87 | 0.89 |

^a^ *F. tularensis* subsp. *tularensis*, ^b^ *F. tularensis* subsp. *mediasiatica*, ^c^ *F. tularensis* subsp. *holarctica*, ^d^ *F. novicida*-like bacterium, *^e^F. novicida*, ^f^*F. novicida*-like bacterium.
