# Supplementary material for: Molecular Evolutionary Consequences of Niche Restriction in Francisella tularensis, a Facultative Intracellular Pathogen
Source: PLoS Pathog. 2009 Jun 12;5(6):e1000472. doi: 10.1371/journal.ppat.1000472 (PMC2688086; doi:10.1371/journal.ppat.1000472)
Supplement: Table S5 — Presence and absence of pseudogenes in different F. tularensis genomes as determined by Psi-Fi with F. novicda U112 (acc. CP000439) as reference. (0.77 MB DOC) [file ppat.1000472.s007.doc]

Table S5. Presence and absence of pseudogenes in different *F. tularensis* genomes as determined by Psi-Fi with *F. novicida* U112 (acc. CP000439) as reference.

| Locus tag^a^ | FSC147^b^ | SCHUS4^c^ | WY-96^d^ | LVS^e^ | FTA^f^ | OSU18^g^ |
| --- | --- | --- | --- | --- | --- | --- |
| FTN_0003 | 1 | 1 | 1 | 1 | 1 | 1 |
| FTN_0022 | 0 | 1 | 1 | 0 | 0 | 0 |
| FTN_0023 | 0 | 0 | 1 | 0 | 0 | 0 |
| FTN_0026 | 0 | 0 | 0 | 1 | 1 | 1 |
| FTN_0027 | 1 | 0 | 0 | 0 | 0 | 0 |
| FTN_0032 | 0 | 0 | 0 | 0 | 0 | 1 |
| FTN_0034 | 1 | 1 | 1 | 0 | 0 | 0 |
| FTN_0055 | 0 | 0 | 0 | 1 | 1 | 1 |
| FTN_0056 | 0 | 1 | 0 | 0 | 0 | 0 |
| FTN_0057 | 1 | 0 | 0 | 1 | 1 | 1 |
| FTN_0058 | 1 | 1 | 0 | 1 | 1 | 0 |
| FTN_0059 | 0 | 0 | 0 | 1 | 1 | 1 |
| FTN_0062 | 1 | 1 | 1 | 1 | 1 | 1 |
| FTN_0063 | 1 | 0 | 0 | 0 | 0 | 0 |
| FTN_0065 | 1 | 1 | 1 | 1 | 1 | 1 |
| FTN_0067 | 1 | 0 | 1 | 1 | 1 | 1 |
| FTN_0079 | 0 | 0 | 0 | 0 | 0 | 1 |
| FTN_0081 | 0 | 1 | 1 | 1 | 1 | 1 |
| FTN_0086 | 1 | 1 | 1 | 1 | 1 | 1 |
| FTN_0087 | 1 | 1 | 0 | 0 | 0 | 0 |
| FTN_0088 | 1 | 0 | 0 | 1 | 1 | 1 |
| FTN_0089 | 1 | 1 | 0 | 0 | 0 | 1 |
| FTN_0090 | 0 | 0 | 0 | 1 | 1 | 1 |
| FTN_0093 | 0 | 1 | 1 | 1 | 1 | 1 |
| FTN_0094 | 1 | 1 | 1 | 1 | 1 | 1 |
| FTN_0100 | 0 | 1 | 0 | 0 | 0 | 0 |
| FTN_0101 | 0 | 0 | 0 | 1 | 1 | 1 |
| FTN_0103 | 1 | 1 | 1 | 1 | 1 | 1 |
| FTN_0115 | 1 | 1 | 1 | 1 | 1 | 1 |
| FTN_0116 | 0 | 0 | 0 | 1 | 1 | 1 |
| FTN_0117 | 1 | 1 | 1 | 1 | 1 | 1 |
| FTN_0127 | 1 | 1 | 1 | 1 | 1 | 1 |
| FTN_0128 | 0 | 0 | 1 | 0 | 0 | 0 |
| FTN_0130 | 1 | 1 | 1 | 1 | 1 | 1 |
| FTN_0131 | 1 | 1 | 1 | 1 | 1 | 1 |
| FTN_0133 | 0 | 0 | 0 | 1 | 1 | 1 |
| FTN_0136 | 0 | 0 | 0 | 1 | 1 | 1 |
| FTN_0138 | 0 | 1 | 1 | 1 | 1 | 1 |
| FTN_0142 | 1 | 1 | 0 | 0 | 0 | 1 |
| FTN_0148 | 1 | 1 | 1 | 1 | 1 | 1 |
| FTN_0149 | 1 | 1 | 1 | 1 | 1 | 1 |
| FTN_0152 | 0 | 1 | 0 | 1 | 1 | 1 |
| FTN_0153 | 1 | 0 | 0 | 1 | 1 | 1 |
| FTN_0154 | 0 | 0 | 0 | 1 | 1 | 1 |
| FTN_0155 | 1 | 1 | 0 | 0 | 0 | 1 |
| FTN_0161 | 1 | 0 | 0 | 0 | 0 | 0 |
| FTN_0170 | 1 | 0 | 0 | 1 | 1 | 1 |
| FTN_0171 | 1 | 0 | 0 | 0 | 0 | 0 |
| FTN_0175 | 1 | 1 | 1 | 1 | 1 | 1 |
| FTN_0176 | 1 | 1 | 1 | 1 | 1 | 1 |
| FTN_0180 | 1 | 1 | 0 | 1 | 1 | 1 |
| FTN_0184 | 0 | 1 | 1 | 1 | 1 | 1 |
| FTN_0188 | 0 | 1 | 0 | 0 | 0 | 0 |
| FTN_0190 | 1 | 1 | 1 | 0 | 0 | 0 |
| FTN_0191 | 0 | 1 | 0 | 0 | 0 | 0 |
| FTN_0200 | 0 | 0 | 0 | 0 | 1 | 0 |
| FTN_0209 | 0 | 0 | 1 | 0 | 1 | 0 |
| FTN_0216 | 0 | 0 | 0 | 1 | 1 | 1 |
| FTN_0219 | 1 | 0 | 0 | 0 | 0 | 0 |
| FTN_0222 | 0 | 0 | 0 | 1 | 1 | 1 |
| FTN_0223 | 1 | 1 | 1 | 1 | 1 | 1 |
| FTN_0225 | 0 | 0 | 1 | 0 | 0 | 0 |
| FTN_0266 | 0 | 0 | 0 | 0 | 0 | 1 |
| FTN_0269 | 1 | 1 | 1 | 1 | 1 | 1 |
| FTN_0280 | 0 | 1 | 1 | 0 | 0 | 0 |
| FTN_0287 | 1 | 1 | 1 | 1 | 1 | 1 |
| FTN_0299 | 1 | 1 | 1 | 1 | 1 | 1 |
| FTN_0301 | 0 | 0 | 0 | 1 | 1 | 1 |
| FTN_0302 | 0 | 1 | 0 | 0 | 0 | 0 |
| FTN_0303 | 1 | 0 | 0 | 0 | 0 | 0 |
| FTN_0304 | 1 | 0 | 0 | 1 | 1 | 1 |
| FTN_0305 | 0 | 1 | 0 | 1 | 1 | 1 |
| FTN_0306 | 1 | 1 | 1 | 1 | 1 | 1 |
| FTN_0307 | 1 | 0 | 0 | 0 | 0 | 0 |
| FTN_0308 | 1 | 1 | 1 | 1 | 1 | 1 |
| FTN_0309 | 1 | 1 | 1 | 1 | 1 | 1 |
| FTN_0312 | 0 | 1 | 1 | 0 | 0 | 0 |
| FTN_0313 | 0 | 1 | 0 | 0 | 0 | 0 |
| FTN_0317 | 0 | 1 | 1 | 1 | 1 | 1 |
| FTN_0319 | 0 | 0 | 0 | 0 | 0 | 1 |
| FTN_0322 | 0 | 0 | 0 | 1 | 1 | 1 |
| FTN_0326 | 0 | 1 | 0 | 0 | 0 | 0 |
| FTN_0336 | 0 | 0 | 1 | 0 | 1 | 0 |
| FTN_0341 | 1 | 0 | 0 | 1 | 1 | 1 |
| FTN_0343 | 0 | 1 | 0 | 1 | 0 | 0 |
| FTN_0344 | 1 | 0 | 0 | 1 | 1 | 1 |
| FTN_0345 | 1 | 1 | 0 | 1 | 1 | 1 |
| FTN_0354 | 0 | 1 | 0 | 0 | 0 | 0 |
| FTN_0358 | 1 | 1 | 1 | 0 | 0 | 0 |
| FTN_0359 | 0 | 1 | 0 | 0 | 0 | 0 |
| FTN_0360 | 0 | 0 | 0 | 1 | 1 | 1 |
| FTN_0361 | 0 | 0 | 0 | 1 | 1 | 1 |
| FTN_0362 | 1 | 1 | 1 | 0 | 0 | 0 |
| FTN_0364 | 1 | 0 | 0 | 1 | 1 | 1 |
| FTN_0365 | 0 | 0 | 0 | 1 | 1 | 1 |
| FTN_0381 | 0 | 1 | 1 | 1 | 0 | 0 |
| FTN_0382 | 1 | 0 | 0 | 1 | 1 | 1 |
| FTN_0389 | 1 | 1 | 1 | 0 | 1 | 1 |
| FTN_0392 | 1 | 0 | 0 | 1 | 1 | 1 |
| FTN_0393 | 0 | 1 | 0 | 1 | 1 | 1 |
| FTN_0394 | 1 | 1 | 1 | 1 | 1 | 1 |
| FTN_0396 | 1 | 0 | 1 | 0 | 0 | 0 |
| FTN_0403 | 0 | 1 | 0 | 0 | 0 | 0 |
| FTN_0406 | 0 | 1 | 0 | 0 | 0 | 0 |
| FTN_0408 | 1 | 1 | 1 | 1 | 1 | 1 |
| FTN_0409 | 0 | 1 | 1 | 1 | 1 | 1 |
| FTN_0411 | 0 | 1 | 0 | 0 | 0 | 0 |
| FTN_0413 | 1 | 1 | 1 | 1 | 1 | 1 |
| FTN_0414 | 0 | 0 | 0 | 1 | 1 | 1 |
| FTN_0424 | 0 | 0 | 0 | 1 | 1 | 1 |
| FTN_0428 | 0 | 0 | 0 | 1 | 1 | 1 |
| FTN_0429 | 1 | 0 | 0 | 0 | 0 | 0 |
| FTN_0437 | 1 | 0 | 0 | 1 | 1 | 1 |
| FTN_0457 | 1 | 1 | 1 | 0 | 0 | 0 |
| FTN_0465 | 0 | 0 | 0 | 1 | 1 | 1 |
| FTN_0468 | 1 | 1 | 1 | 1 | 1 | 1 |
| FTN_0471 | 1 | 1 | 0 | 0 | 0 | 0 |
| FTN_0477 | 1 | 0 | 0 | 1 | 1 | 1 |
| FTN_0486 | 1 | 0 | 1 | 0 | 0 | 0 |
| FTN_0497 | 0 | 1 | 1 | 1 | 1 | 1 |
| FTN_0498 | 1 | 1 | 1 | 1 | 1 | 1 |
| FTN_0500 | 1 | 0 | 0 | 0 | 0 | 0 |
| FTN_0504 | 1 | 0 | 0 | 0 | 0 | 0 |
| FTN_0515 | 0 | 1 | 0 | 0 | 0 | 0 |
| FTN_0521 | 0 | 1 | 0 | 0 | 0 | 0 |
| FTN_0523 | 0 | 1 | 0 | 0 | 0 | 0 |
| FTN_0524 | 1 | 0 | 0 | 0 | 0 | 1 |
| FTN_0525 | 0 | 1 | 0 | 1 | 1 | 1 |
| FTN_0526 | 0 | 1 | 0 | 1 | 1 | 1 |
| FTN_0527 | 1 | 0 | 0 | 0 | 0 | 0 |
| FTN_0531 | 1 | 0 | 1 | 1 | 1 | 1 |
| FTN_0532 | 1 | 1 | 0 | 0 | 0 | 0 |
| FTN_0533 | 1 | 0 | 0 | 0 | 1 | 0 |
| FTN_0536 | 0 | 1 | 0 | 0 | 0 | 0 |
| FTN_0537 | 1 | 0 | 1 | 1 | 1 | 1 |
| FTN_0538 | 1 | 1 | 1 | 1 | 1 | 1 |
| FTN_0555 | 1 | 0 | 0 | 0 | 0 | 0 |
| FTN_0556 | 1 | 1 | 0 | 1 | 1 | 1 |
| FTN_0566 | 0 | 0 | 0 | 1 | 1 | 1 |
| FTN_0576 | 0 | 0 | 0 | 1 | 1 | 1 |
| FTN_0578 | 1 | 1 | 1 | 1 | 1 | 1 |
| FTN_0579 | 0 | 1 | 0 | 1 | 1 | 1 |
| FTN_0581 | 1 | 0 | 0 | 0 | 1 | 1 |
| FTN_0583 | 1 | 0 | 0 | 0 | 0 | 0 |
| FTN_0584 | 1 | 1 | 1 | 0 | 0 | 0 |
| FTN_0586 | 0 | 0 | 1 | 1 | 1 | 1 |
| FTN_0588 | 0 | 1 | 1 | 1 | 0 | 0 |
| FTN_0589 | 0 | 1 | 1 | 1 | 1 | 1 |
| FTN_0590 | 0 | 1 | 0 | 1 | 1 | 0 |
| FTN_0591 | 0 | 0 | 0 | 0 | 1 | 0 |
| FTN_0596 | 1 | 1 | 1 | 1 | 1 | 1 |
| FTN_0617 | 0 | 1 | 0 | 1 | 1 | 1 |
| FTN_0618 | 0 | 1 | 1 | 1 | 1 | 1 |
| FTN_0624 | 0 | 0 | 0 | 0 | 0 | 1 |
| FTN_0627 | 0 | 0 | 1 | 0 | 0 | 0 |
| FTN_0629 | 1 | 1 | 1 | 1 | 1 | 1 |
| FTN_0634 | 1 | 0 | 0 | 1 | 1 | 1 |
| FTN_0635 | 1 | 1 | 0 | 1 | 0 | 0 |
| FTN_0638 | 1 | 0 | 0 | 0 | 0 | 0 |
| FTN_0644 | 0 | 0 | 0 | 0 | 1 | 0 |
| FTN_0649 | 0 | 1 | 0 | 0 | 0 | 0 |
| FTN_0667 | 1 | 0 | 0 | 1 | 1 | 1 |
| FTN_0671 | 0 | 0 | 1 | 0 | 0 | 0 |
| FTN_0673 | 1 | 1 | 1 | 1 | 1 | 1 |
| FTN_0674 | 1 | 1 | 1 | 1 | 1 | 1 |
| FTN_0678 | 0 | 1 | 0 | 0 | 0 | 0 |
| FTN_0681 | 1 | 1 | 1 | 1 | 1 | 1 |
| FTN_0695 | 1 | 1 | 1 | 1 | 1 | 1 |
| FTN_0696 | 1 | 1 | 1 | 1 | 1 | 1 |
| FTN_0699 | 1 | 1 | 1 | 1 | 1 | 1 |
| FTN_0700 | 1 | 1 | 1 | 1 | 1 | 1 |
| FTN_0701 | 0 | 0 | 0 | 0 | 0 | 1 |
| FTN_0713 | 1 | 1 | 1 | 1 | 1 | 1 |
| FTN_0714 | 1 | 0 | 0 | 1 | 1 | 1 |
| FTN_0715 | 1 | 0 | 0 | 1 | 1 | 1 |
| FTN_0717 | 1 | 1 | 1 | 1 | 1 | 1 |
| FTN_0718 | 0 | 0 | 0 | 1 | 1 | 1 |
| FTN_0721 | 1 | 0 | 0 | 0 | 1 | 0 |
| FTN_0724 | 1 | 1 | 1 | 1 | 1 | 1 |
| FTN_0727 | 1 | 0 | 0 | 0 | 1 | 0 |
| FTN_0730 | 1 | 1 | 1 | 1 | 1 | 1 |
| FTN_0740 | 0 | 0 | 0 | 1 | 1 | 1 |
| FTN_0741 | 0 | 1 | 1 | 1 | 1 | 1 |
| FTN_0742 | 0 | 0 | 0 | 1 | 1 | 1 |
| FTN_0747 | 1 | 1 | 0 | 1 | 1 | 1 |
| FTN_0757 | 1 | 1 | 1 | 1 | 1 | 1 |
| FTN_0758 | 1 | 1 | 1 | 1 | 1 | 1 |
| FTN_0759 | 0 | 1 | 0 | 0 | 0 | 0 |
| FTN_0760 | 1 | 0 | 0 | 0 | 0 | 0 |
| FTN_0765 | 0 | 0 | 0 | 1 | 1 | 1 |
| FTN_0767 | 0 | 1 | 1 | 1 | 1 | 1 |
| FTN_0768 | 1 | 0 | 0 | 0 | 0 | 0 |
| FTN_0770 | 1 | 1 | 1 | 1 | 1 | 1 |
| FTN_0771 | 0 | 1 | 0 | 0 | 0 | 0 |
| FTN_0773 | 1 | 1 | 1 | 1 | 1 | 1 |
| FTN_0776 | 1 | 1 | 0 | 1 | 1 | 1 |
| FTN_0782 | 1 | 1 | 1 | 1 | 1 | 1 |
| FTN_0783 | 0 | 0 | 0 | 1 | 1 | 1 |
| FTN_0788 | 1 | 0 | 0 | 0 | 0 | 0 |
| FTN_0793 | 0 | 0 | 0 | 0 | 1 | 1 |
| FTN_0807 | 1 | 1 | 1 | 1 | 1 | 1 |
| FTN_0808 | 1 | 1 | 1 | 1 | 1 | 1 |
| FTN_0809 | 1 | 0 | 1 | 0 | 0 | 1 |
| FTN_0810 | 1 | 1 | 1 | 0 | 0 | 0 |
| FTN_0811 | 1 | 1 | 0 | 1 | 1 | 1 |
| FTN_0812 | 0 | 0 | 0 | 1 | 1 | 1 |
| FTN_0824 | 1 | 1 | 1 | 1 | 1 | 1 |
| FTN_0825 | 0 | 0 | 1 | 1 | 0 | 0 |
| FTN_0826 | 1 | 0 | 1 | 1 | 0 | 0 |
| FTN_0827 | 0 | 0 | 0 | 1 | 1 | 1 |
| FTN_0829 | 1 | 1 | 1 | 1 | 1 | 1 |
| FTN_0836 | 0 | 0 | 1 | 0 | 0 | 0 |
| FTN_0839 | 1 | 1 | 1 | 1 | 1 | 1 |
| FTN_0840 | 1 | 0 | 0 | 0 | 0 | 0 |
| FTN_0841 | 1 | 0 | 0 | 1 | 1 | 1 |
| FTN_0845 | 0 | 0 | 1 | 0 | 0 | 0 |
| FTN_0847 | 1 | 1 | 1 | 1 | 1 | 1 |
| FTN_0854 | 1 | 1 | 1 | 1 | 1 | 1 |
| FTN_0855 | 0 | 0 | 1 | 0 | 0 | 0 |
| FTN_0857 | 1 | 1 | 1 | 1 | 1 | 1 |
| FTN_0861 | 0 | 0 | 0 | 1 | 1 | 1 |
| FTN_0863 | 1 | 1 | 1 | 0 | 0 | 0 |
| FTN_0865 | 1 | 1 | 1 | 1 | 1 | 1 |
| FTN_0868 | 1 | 1 | 1 | 1 | 1 | 1 |
| FTN_0869 | 0 | 0 | 1 | 0 | 0 | 0 |
| FTN_0872 | 0 | 0 | 0 | 1 | 1 | 1 |
| FTN_0875 | 1 | 0 | 0 | 1 | 1 | 1 |
| FTN_0876 | 1 | 1 | 1 | 0 | 0 | 0 |
| FTN_0879 | 0 | 1 | 0 | 0 | 0 | 0 |
| FTN_0884 | 0 | 0 | 0 | 0 | 1 | 0 |
| FTN_0885 | 0 | 0 | 0 | 1 | 1 | 1 |
| FTN_0886 | 1 | 1 | 0 | 1 | 1 | 1 |
| FTN_0887 | 1 | 1 | 0 | 1 | 0 | 1 |
| FTN_0888 | 0 | 0 | 0 | 1 | 1 | 1 |
| FTN_0890 | 1 | 1 | 1 | 1 | 1 | 1 |
| FTN_0898 | 0 | 0 | 0 | 1 | 1 | 1 |
| FTN_0910 | 1 | 1 | 1 | 1 | 1 | 1 |
| FTN_0911 | 1 | 1 | 1 | 1 | 1 | 1 |
| FTN_0912 | 1 | 0 | 0 | 0 | 0 | 0 |
| FTN_0925 | 1 | 1 | 1 | 1 | 1 | 1 |
| FTN_0926 | 0 | 1 | 1 | 1 | 1 | 1 |
| FTN_0927 | 1 | 0 | 0 | 1 | 1 | 1 |
| FTN_0940 | 1 | 0 | 0 | 0 | 0 | 0 |
| FTN_0941 | 1 | 1 | 1 | 1 | 1 | 1 |
| FTN_0952 | 1 | 1 | 1 | 0 | 0 | 0 |
| FTN_0954 | 1 | 1 | 0 | 0 | 0 | 0 |
| FTN_0962 | 0 | 0 | 0 | 1 | 1 | 1 |
| FTN_0963 | 1 | 0 | 0 | 1 | 1 | 1 |
| FTN_0964 | 1 | 1 | 1 | 1 | 1 | 1 |
| FTN_0969 | 1 | 1 | 1 | 1 | 1 | 1 |
| FTN_0971 | 0 | 0 | 0 | 1 | 1 | 1 |
| FTN_0972 | 1 | 0 | 0 | 1 | 1 | 1 |
| FTN_0973 | 0 | 1 | 0 | 0 | 0 | 0 |
| FTN_0976 | 1 | 1 | 1 | 1 | 1 | 1 |
| FTN_0977 | 1 | 0 | 0 | 0 | 0 | 0 |
| FTN_0984 | 1 | 1 | 1 | 0 | 0 | 0 |
| FTN_0986 | 0 | 1 | 1 | 0 | 0 | 0 |
| FTN_0989 | 0 | 1 | 1 | 0 | 0 | 0 |
| FTN_0990 | 1 | 1 | 1 | 1 | 1 | 1 |
| FTN_0991 | 0 | 1 | 0 | 0 | 0 | 0 |
| FTN_0997 | 1 | 1 | 0 | 0 | 0 | 0 |
| FTN_0998 | 0 | 1 | 1 | 1 | 1 | 1 |
| FTN_1000 | 1 | 1 | 1 | 1 | 1 | 1 |
| FTN_1005 | 0 | 0 | 1 | 0 | 0 | 0 |
| FTN_1009 | 1 | 0 | 0 | 1 | 1 | 1 |
| FTN_1010 | 1 | 1 | 0 | 1 | 1 | 1 |
| FTN_1011 | 0 | 0 | 0 | 1 | 1 | 1 |
| FTN_1012 | 1 | 1 | 1 | 1 | 1 | 1 |
| FTN_1018 | 0 | 1 | 0 | 0 | 0 | 0 |
| FTN_1021 | 1 | 1 | 1 | 1 | 1 | 1 |
| FTN_1026 | 0 | 1 | 0 | 0 | 0 | 0 |
| FTN_1031 | 0 | 0 | 1 | 0 | 0 | 0 |
| FTN_1032 | 0 | 0 | 0 | 1 | 1 | 1 |
| FTN_1040 | 1 | 1 | 0 | 0 | 0 | 0 |
| FTN_1042 | 1 | 1 | 1 | 1 | 1 | 1 |
| FTN_1043 | 0 | 1 | 0 | 1 | 1 | 1 |
| FTN_1044 | 1 | 1 | 1 | 1 | 1 | 1 |
| FTN_1049 | 0 | 0 | 0 | 1 | 1 | 1 |
| FTN_1061 | 1 | 0 | 0 | 1 | 1 | 1 |
| FTN_1068 | 0 | 0 | 0 | 1 | 1 | 1 |
| FTN_1069 | 1 | 1 | 1 | 1 | 1 | 1 |
| FTN_1070 | 1 | 1 | 1 | 1 | 1 | 1 |
| FTN_1071 | 1 | 1 | 1 | 1 | 1 | 1 |
| FTN_1075 | 0 | 1 | 0 | 0 | 0 | 0 |
| FTN_1077 | 1 | 1 | 1 | 1 | 1 | 1 |
| FTN_1098 | 1 | 0 | 0 | 1 | 1 | 1 |
| FTN_1099 | 1 | 0 | 0 | 1 | 1 | 1 |
| FTN_1104 | 1 | 1 | 1 | 1 | 1 | 1 |
| FTN_1106 | 1 | 0 | 0 | 0 | 0 | 0 |
| FTN_1108 | 0 | 0 | 0 | 1 | 1 | 1 |
| FTN_1113 | 1 | 0 | 0 | 0 | 0 | 0 |
| FTN_1117 | 1 | 1 | 1 | 1 | 1 | 1 |
| FTN_1121 | 1 | 1 | 0 | 1 | 1 | 1 |
| FTN_1123 | 1 | 0 | 1 | 0 | 1 | 1 |
| FTN_1125 | 1 | 1 | 1 | 1 | 1 | 1 |
| FTN_1126 | 0 | 1 | 0 | 0 | 0 | 0 |
| FTN_1127 | 1 | 1 | 1 | 1 | 1 | 1 |
| FTN_1134 | 0 | 0 | 1 | 1 | 1 | 1 |
| FTN_1143 | 1 | 1 | 1 | 1 | 1 | 1 |
| FTN_1144 | 0 | 0 | 0 | 1 | 1 | 1 |
| FTN_1152 | 0 | 0 | 0 | 1 | 1 | 1 |
| FTN_1155 | 1 | 1 | 1 | 1 | 1 | 1 |
| FTN_1156 | 1 | 1 | 1 | 1 | 1 | 0 |
| FTN_1158 | 0 | 1 | 1 | 0 | 0 | 0 |
| FTN_1160 | 0 | 1 | 1 | 0 | 0 | 0 |
| FTN_1166 | 1 | 1 | 1 | 1 | 1 | 1 |
| FTN_1169 | 1 | 0 | 0 | 1 | 1 | 1 |
| FTN_1170 | 1 | 1 | 0 | 0 | 0 | 1 |
| FTN_1171 | 1 | 1 | 0 | 1 | 1 | 1 |
| FTN_1172 | 1 | 1 | 0 | 1 | 1 | 1 |
| FTN_1179 | 0 | 0 | 0 | 1 | 1 | 1 |
| FTN_1186 | 1 | 1 | 0 | 1 | 1 | 1 |
| FTN_1192 | 1 | 1 | 0 | 1 | 1 | 1 |
| FTN_1193 | 0 | 0 | 0 | 1 | 0 | 0 |
| FTN_1223 | 1 | 0 | 0 | 0 | 0 | 0 |
| FTN_1224 | 0 | 0 | 0 | 1 | 1 | 1 |
| FTN_1225 | 0 | 0 | 0 | 0 | 0 | 1 |
| FTN_1226 | 1 | 0 | 0 | 0 | 0 | 0 |
| FTN_1227 | 0 | 0 | 0 | 1 | 1 | 1 |
| FTN_1230 | 1 | 1 | 1 | 1 | 1 | 1 |
| FTN_1231 | 1 | 0 | 0 | 0 | 0 | 0 |
| FTN_1251 | 0 | 1 | 1 | 1 | 1 | 1 |
| FTN_1252 | 0 | 0 | 0 | 1 | 1 | 1 |
| FTN_1260 | 1 | 0 | 0 | 0 | 0 | 1 |
| FTN_1262 | 1 | 0 | 1 | 1 | 1 | 1 |
| FTN_1265 | 1 | 0 | 0 | 0 | 0 | 0 |
| FTN_1272 | 0 | 0 | 0 | 1 | 1 | 0 |
| FTN_1280 | 1 | 1 | 1 | 1 | 1 | 1 |
| FTN_1281 | 1 | 0 | 0 | 1 | 1 | 1 |
| FTN_1292 | 1 | 0 | 0 | 0 | 0 | 0 |
| FTN_1300 | 1 | 0 | 0 | 1 | 1 | 1 |
| FTN_1301 | 1 | 1 | 1 | 1 | 1 | 1 |
| FTN_1309 | 1 | 0 | 0 | 0 | 0 | 0 |
| FTN_1313 | 1 | 0 | 0 | 1 | 1 | 1 |
| FTN_1325 | 0 | 0 | 0 | 1 | 1 | 1 |
| FTN_1326 | 1 | 1 | 1 | 0 | 0 | 0 |
| FTN_1328 | 1 | 1 | 1 | 1 | 1 | 1 |
| FTN_1342 | 0 | 1 | 0 | 0 | 0 | 0 |
| FTN_1344 | 0 | 1 | 1 | 0 | 0 | 0 |
| FTN_1345 | 1 | 1 | 0 | 0 | 0 | 0 |
| FTN_1347 | 0 | 0 | 0 | 1 | 1 | 1 |
| FTN_1349 | 1 | 1 | 1 | 1 | 1 | 1 |
| FTN_1354 | 0 | 0 | 0 | 1 | 0 | 0 |
| FTN_1360 | 0 | 1 | 0 | 0 | 0 | 0 |
| FTN_1361 | 0 | 0 | 1 | 0 | 0 | 0 |
| FTN_1362 | 0 | 0 | 0 | 1 | 1 | 1 |
| FTN_1376 | 0 | 0 | 0 | 0 | 0 | 1 |
| FTN_1380 | 0 | 0 | 0 | 1 | 1 | 1 |
| FTN_1381 | 1 | 1 | 1 | 1 | 1 | 1 |
| FTN_1385 | 0 | 1 | 0 | 0 | 0 | 0 |
| FTN_1386 | 0 | 0 | 0 | 1 | 1 | 1 |
| FTN_1389 | 1 | 0 | 0 | 0 | 0 | 0 |
| FTN_1391 | 1 | 0 | 1 | 1 | 1 | 1 |
| FTN_1392 | 1 | 0 | 0 | 0 | 1 | 1 |
| FTN_1398 | 0 | 0 | 0 | 1 | 1 | 1 |
| FTN_1399 | 0 | 1 | 0 | 0 | 0 | 0 |
| FTN_1400 | 1 | 1 | 1 | 0 | 0 | 0 |
| FTN_1406 | 0 | 1 | 0 | 0 | 0 | 0 |
| FTN_1407 | 0 | 1 | 0 | 0 | 0 | 0 |
| FTN_1409 | 1 | 1 | 1 | 1 | 1 | 1 |
| FTN_1413 | 1 | 1 | 1 | 1 | 1 | 1 |
| FTN_1432 | 0 | 0 | 1 | 0 | 0 | 0 |
| FTN_1441 | 1 | 1 | 1 | 0 | 0 | 0 |
| FTN_1442 | 0 | 1 | 0 | 0 | 0 | 0 |
| FTN_1444 | 1 | 1 | 1 | 1 | 1 | 1 |
| FTN_1445 | 0 | 0 | 0 | 1 | 1 | 1 |
| FTN_1450 | 0 | 1 | 0 | 0 | 0 | 0 |
| FTN_1452 | 0 | 0 | 0 | 1 | 1 | 1 |
| FTN_1453 | 1 | 1 | 0 | 1 | 1 | 1 |
| FTN_1454 | 1 | 1 | 0 | 0 | 0 | 0 |
| FTN_1455 | 1 | 1 | 1 | 1 | 1 | 1 |
| FTN_1456 | 1 | 1 | 1 | 0 | 0 | 0 |
| FTN_1457 | 1 | 1 | 0 | 0 | 0 | 0 |
| FTN_1458 | 1 | 0 | 0 | 0 | 0 | 0 |
| FTN_1460 | 1 | 0 | 0 | 1 | 1 | 1 |
| FTN_1466 | 1 | 1 | 0 | 1 | 1 | 1 |
| FTN_1467 | 1 | 0 | 0 | 0 | 0 | 0 |
| FTN_1474 | 1 | 1 | 1 | 1 | 0 | 1 |
| FTN_1475 | 0 | 1 | 0 | 0 | 0 | 0 |
| FTN_1485 | 1 | 1 | 1 | 1 | 1 | 1 |
| FTN_1487 | 0 | 0 | 1 | 0 | 0 | 0 |
| FTN_1491 | 1 | 1 | 1 | 1 | 1 | 1 |
| FTN_1495 | 1 | 1 | 1 | 1 | 1 | 1 |
| FTN_1502 | 1 | 1 | 1 | 1 | 1 | 1 |
| FTN_1511 | 1 | 1 | 1 | 1 | 1 | 1 |
| FTN_1515 | 0 | 0 | 1 | 0 | 0 | 0 |
| FTN_1516 | 1 | 0 | 0 | 0 | 0 | 0 |
| FTN_1519 | 0 | 0 | 0 | 0 | 1 | 0 |
| FTN_1520 | 1 | 0 | 0 | 0 | 0 | 0 |
| FTN_1523 | 1 | 0 | 0 | 0 | 0 | 0 |
| FTN_1524 | 1 | 0 | 0 | 1 | 1 | 1 |
| FTN_1526 | 0 | 1 | 0 | 0 | 0 | 0 |
| FTN_1529 | 1 | 1 | 1 | 1 | 1 | 1 |
| FTN_1530 | 1 | 1 | 0 | 0 | 0 | 0 |
| FTN_1531 | 0 | 1 | 0 | 1 | 1 | 1 |
| FTN_1532 | 0 | 0 | 0 | 0 | 0 | 1 |
| FTN_1533 | 0 | 1 | 1 | 1 | 0 | 1 |
| FTN_1536 | 0 | 0 | 0 | 1 | 1 | 1 |
| FTN_1543 | 0 | 0 | 1 | 0 | 0 | 0 |
| FTN_1554 | 1 | 1 | 1 | 1 | 0 | 0 |
| FTN_1555 | 1 | 0 | 0 | 0 | 0 | 0 |
| FTN_1557 | 1 | 0 | 0 | 0 | 0 | 0 |
| FTN_1567 | 0 | 0 | 0 | 0 | 1 | 0 |
| FTN_1574 | 0 | 0 | 0 | 0 | 0 | 1 |
| FTN_1580 | 1 | 0 | 0 | 0 | 0 | 0 |
| FTN_1586 | 0 | 0 | 0 | 1 | 1 | 1 |
| FTN_1587 | 1 | 1 | 1 | 0 | 0 | 0 |
| FTN_1589 | 1 | 0 | 1 | 0 | 0 | 0 |
| FTN_1590 | 0 | 0 | 0 | 1 | 1 | 1 |
| FTN_1591 | 1 | 1 | 1 | 1 | 1 | 1 |
| FTN_1592 | 1 | 1 | 1 | 1 | 1 | 1 |
| FTN_1593 | 1 | 0 | 0 | 1 | 1 | 1 |
| FTN_1615 | 1 | 1 | 1 | 0 | 1 | 0 |
| FTN_1616 | 1 | 0 | 0 | 1 | 1 | 1 |
| FTN_1619 | 1 | 1 | 1 | 1 | 1 | 1 |
| FTN_1620 | 1 | 0 | 1 | 0 | 0 | 0 |
| FTN_1622 | 1 | 0 | 0 | 1 | 1 | 1 |
| FTN_1628 | 1 | 1 | 1 | 1 | 1 | 1 |
| FTN_1665 | 1 | 1 | 1 | 1 | 1 | 1 |
| FTN_1687 | 1 | 1 | 1 | 1 | 1 | 1 |
| FTN_1692 | 0 | 0 | 0 | 1 | 1 | 1 |
| FTN_1693 | 0 | 0 | 0 | 1 | 1 | 1 |
| FTN_1695 | 1 | 1 | 1 | 1 | 1 | 1 |
| FTN_1696 | 1 | 0 | 0 | 0 | 0 | 0 |
| FTN_1697 | 0 | 1 | 0 | 0 | 0 | 0 |
| FTN_1701 | 1 | 0 | 0 | 1 | 1 | 1 |
| FTN_1708 | 1 | 1 | 1 | 1 | 1 | 1 |
| FTN_1712 | 1 | 1 | 1 | 1 | 1 | 1 |
| FTN_1713 | 0 | 1 | 0 | 1 | 1 | 1 |
| FTN_1714 | 1 | 1 | 1 | 1 | 1 | 1 |
| FTN_1715 | 0 | 0 | 0 | 1 | 1 | 1 |
| FTN_1717 | 0 | 0 | 0 | 1 | 1 | 1 |
| FTN_1718 | 0 | 1 | 0 | 0 | 0 | 0 |
| FTN_1719 | 0 | 1 | 1 | 1 | 1 | 1 |
| FTN_1733 | 1 | 1 | 1 | 0 | 0 | 0 |
| FTN_1736 | 1 | 0 | 0 | 1 | 1 | 1 |
| FTN_1737 | 0 | 0 | 0 | 1 | 0 | 0 |
| FTN_1738 | 1 | 1 | 0 | 0 | 0 | 0 |
| FTN_1741 | 1 | 0 | 0 | 0 | 0 | 0 |
| FTN_1742 | 1 | 1 | 1 | 1 | 1 | 1 |
| FTN_1744 | 0 | 1 | 1 | 0 | 0 | 0 |
| FTN_1755 | 1 | 1 | 0 | 1 | 1 | 1 |
| FTN_1755 | 0 | 1 | 1 | 1 | 1 | 1 |
| FTN_1764 | 1 | 0 | 0 | 0 | 0 | 0 |
| FTN_1765 | 0 | 1 | 0 | 0 | 0 | 0 |
| FTN_1768 | 0 | 0 | 1 | 0 | 0 | 0 |
| FTN_1771 | 0 | 0 | 0 | 1 | 1 | 1 |
| FTN_1772 | 1 | 0 | 0 | 0 | 0 | 0 |
| FTN_1773 | 1 | 0 | 0 | 0 | 0 | 0 |
| FTN_1774 | 0 | 1 | 0 | 0 | 0 | 0 |

^a^Corresponds to locus tags in the *F. novicida* U112 annotation, GenBank acc. no. CP000439. ^b^*F. tularensis* subsp. *mediasiatica* FSC147 (acc. CP000915), ^c^*F. tularensis* subsp. *tularensis* A1 SCHUS4 (acc. AJ749949), ^d^*F. tularensis* subsp. *tularensis* A2 WY96-3418 (acc. CP000608), ^e^ *F. tularensis* subsp. *holarctica* LVS (acc. AM233362), ^f^ *F. tularensis* subsp. *holarctica* FTA (acc. CP000803), ^g^*F. tularensis* subsp. *holarctica* OSU18 (acc.CP000437).
