# Supplementary material for: Molecular Evolutionary Consequences of Niche Restriction in Francisella tularensis, a Facultative Intracellular Pathogen
Source: PLoS Pathog. 2009 Jun 12;5(6):e1000472. doi: 10.1371/journal.ppat.1000472 (PMC2688086; doi:10.1371/journal.ppat.1000472)
Supplement: Table S6 — Missing genes in different F. tularensis genomes as compared to F. novicida U112 (GenBank acc. no. CP000439). Zero denotes the absence and one the presence of a gene. (0.31 MB DOC) [file ppat.1000472.s008.doc]

Table S6. Missing genes in different *F. tularensis* genomes as compared to *F. novicida* U112 (GenBank acc. no. CP000439). Zero denotes the absence and one the presence of a gene.

| Locus tag | FSC147^a^ | SCHUS4^b^ | WY-96^c^ | LVS^d^ | FTA^e^ | OSU18^f^ |
| --- | --- | --- | --- | --- | --- | --- |
| FTN_0005 | 0 | 0 | 0 | 0 | 1 | 0 |
| FTN_0006 | 1 | 0 | 0 | 0 | 0 | 0 |
| FTN_0007 | 1 | 1 | 1 | 0 | 0 | 0 |
| FTN_0008 | 1 | 1 | 1 | 0 | 0 | 0 |
| FTN_0010 | 1 | 1 | 1 | 0 | 0 | 0 |
| FTN_0011 | 0 | 0 | 0 | 1 | 1 | 1 |
| FTN_0012 | 0 | 0 | 0 | 1 | 1 | 1 |
| FTN_0013 | 0 | 0 | 0 | 1 | 1 | 1 |
| FTN_0014 | 1 | 0 | 0 | 1 | 1 | 1 |
| FTN_0015 | 1 | 1 | 1 | 1 | 1 | 1 |
| FTN_0016 | 1 | 1 | 1 | 1 | 1 | 1 |
| FTN_0017 | 1 | 1 | 1 | 1 | 1 | 1 |
| FTN_0025 | 1 | 1 | 1 | 1 | 1 | 1 |
| FTN_0038 | 1 | 1 | 1 | 1 | 1 | 1 |
| FTN_0039 | 1 | 1 | 1 | 1 | 1 | 1 |
| FTN_0040 | 1 | 1 | 1 | 1 | 1 | 1 |
| FTN_0041 | 1 | 1 | 1 | 1 | 1 | 1 |
| FTN_0044 | 1 | 1 | 1 | 1 | 1 | 1 |
| FTN_0045 | 1 | 1 | 1 | 1 | 1 | 1 |
| FTN_0046 | 1 | 1 | 1 | 1 | 1 | 1 |
| FTN_0047 | 1 | 1 | 1 | 1 | 1 | 1 |
| FTN_0048 | 1 | 1 | 1 | 1 | 1 | 1 |
| FTN_0049 | 1 | 1 | 1 | 1 | 1 | 1 |
| FTN_0050 | 1 | 1 | 1 | 1 | 1 | 1 |
| FTN_0052 | 1 | 1 | 1 | 1 | 1 | 1 |
| FTN_0053 | 1 | 1 | 1 | 1 | 1 | 1 |
| FTN_0054 | 1 | 1 | 1 | 1 | 1 | 1 |
| FTN_0055 | 1 | 1 | 1 | 1 | 1 | 1 |
| FTN_0059 | 1 | 1 | 1 | 1 | 1 | 1 |
| FTN_0060 | 1 | 1 | 1 | 1 | 1 | 1 |
| FTN_0147 | 1 | 1 | 1 | 1 | 1 | 1 |
| FTN_0215 | 1 | 1 | 1 | 1 | 1 | 1 |
| FTN_0224 | 1 | 1 | 1 | 1 | 1 | 1 |
| FTN_0260 | 1 | 1 | 1 | 1 | 1 | 1 |
| FTN_0267 | 1 | 1 | 1 | 1 | 1 | 1 |
| FTN_0268 | 1 | 1 | 1 | 1 | 1 | 1 |
| FTN_0282 | 1 | 1 | 1 | 1 | 1 | 1 |
| FTN_0283 | 1 | 1 | 1 | 1 | 1 | 1 |
| FTN_0284 | 1 | 1 | 1 | 1 | 1 | 1 |
| FTN_0285 | 1 | 1 | 1 | 1 | 1 | 1 |
| FTN_0286 | 1 | 1 | 1 | 1 | 1 | 1 |
| FTN_0288 | 1 | 1 | 1 | 1 | 1 | 1 |
| FTN_0314 | 1 | 1 | 1 | 1 | 1 | 1 |
| FTN_0315 | 1 | 1 | 1 | 1 | 1 | 1 |
| FTN_0366 | 1 | 1 | 1 | 1 | 1 | 1 |
| FTN_0367 | 1 | 1 | 1 | 1 | 1 | 1 |
| FTN_0368 | 1 | 1 | 1 | 1 | 1 | 1 |
| FTN_0369 | 1 | 1 | 1 | 1 | 1 | 1 |
| FTN_0370 | 1 | 1 | 1 | 1 | 1 | 1 |
| FTN_0371 | 1 | 1 | 1 | 1 | 1 | 1 |
| FTN_0372 | 1 | 1 | 1 | 1 | 1 | 1 |
| FTN_0373 | 1 | 1 | 1 | 1 | 1 | 1 |
| FTN_0374 | 1 | 1 | 1 | 1 | 1 | 1 |
| FTN_0375 | 1 | 1 | 1 | 1 | 1 | 1 |
| FTN_0376 | 1 | 1 | 1 | 1 | 1 | 1 |
| FTN_0377 | 1 | 1 | 1 | 1 | 1 | 1 |
| FTN_0378 | 1 | 1 | 1 | 1 | 1 | 1 |
| FTN_0379 | 1 | 1 | 1 | 1 | 1 | 1 |
| FTN_0380 | 1 | 1 | 1 | 1 | 1 | 1 |
| FTN_0451 | 1 | 1 | 1 | 1 | 1 | 1 |
| FTN_0452 | 1 | 1 | 1 | 1 | 1 | 1 |
| FTN_0453 | 1 | 1 | 1 | 1 | 1 | 1 |
| FTN_0454 | 1 | 1 | 1 | 1 | 1 | 1 |
| FTN_0455 | 1 | 1 | 1 | 1 | 1 | 1 |
| FTN_0456 | 1 | 1 | 1 | 1 | 1 | 1 |
| FTN_0469 | 1 | 1 | 1 | 1 | 1 | 1 |
| FTN_0470 | 1 | 1 | 1 | 1 | 1 | 1 |
| FTN_0472 | 1 | 1 | 1 | 1 | 1 | 1 |
| FTN_0473 | 1 | 1 | 1 | 1 | 1 | 1 |
| FTN_0474 | 1 | 1 | 1 | 1 | 1 | 1 |
| FTN_0475 | 1 | 1 | 1 | 1 | 1 | 1 |
| FTN_0476 | 1 | 1 | 1 | 1 | 1 | 1 |
| FTN_0501 | 1 | 1 | 1 | 1 | 1 | 1 |
| FTN_0509 | 1 | 1 | 1 | 1 | 1 | 1 |
| FTN_0510 | 1 | 1 | 1 | 1 | 1 | 1 |
| FTN_0614 | 1 | 1 | 1 | 1 | 1 | 1 |
| FTN_0619 | 1 | 1 | 1 | 1 | 1 | 1 |
| FTN_0650 | 1 | 1 | 1 | 1 | 1 | 1 |
| FTN_0703 | 1 | 1 | 1 | 1 | 1 | 1 |
| FTN_0705 | 1 | 1 | 1 | 1 | 1 | 1 |
| FTN_0706 | 1 | 1 | 1 | 1 | 1 | 1 |
| FTN_0707 | 1 | 1 | 1 | 1 | 1 | 1 |
| FTN_0708 | 1 | 1 | 1 | 1 | 1 | 1 |
| FTN_0709 | 1 | 1 | 1 | 1 | 1 | 1 |
| FTN_0726 | 1 | 1 | 1 | 1 | 1 | 1 |
| FTN_0761 | 1 | 1 | 1 | 1 | 1 | 1 |
| FTN_0763 | 1 | 1 | 1 | 1 | 1 | 1 |
| FTN_0784 | 1 | 1 | 1 | 1 | 1 | 1 |
| FTN_0794 | 1 | 1 | 1 | 1 | 1 | 1 |
| FTN_0796 | 1 | 1 | 1 | 1 | 1 | 1 |
| FTN_0799 | 1 | 1 | 1 | 1 | 1 | 1 |
| FTN_0800 | 1 | 1 | 1 | 1 | 1 | 1 |
| FTN_0801 | 1 | 1 | 1 | 1 | 1 | 1 |
| FTN_0837 | 1 | 1 | 1 | 1 | 1 | 1 |
| FTN_0840 | 1 | 1 | 1 | 1 | 1 | 1 |
| FTN_0863 | 1 | 1 | 1 | 1 | 1 | 1 |
| FTN_0887 | 1 | 1 | 1 | 1 | 1 | 1 |
| FTN_0928 | 1 | 1 | 1 | 1 | 1 | 1 |
| FTN_0929 | 1 | 1 | 1 | 1 | 1 | 1 |
| FTN_0930 | 1 | 1 | 1 | 1 | 1 | 1 |
| FTN_0931 | 1 | 1 | 1 | 1 | 1 | 1 |
| FTN_0933 | 1 | 1 | 1 | 1 | 1 | 1 |
| FTN_0934 | 1 | 1 | 1 | 1 | 1 | 1 |
| FTN_0935 | 1 | 1 | 1 | 1 | 1 | 1 |
| FTN_0936 | 1 | 1 | 1 | 1 | 1 | 1 |
| FTN_0937 | 1 | 1 | 1 | 1 | 1 | 1 |
| FTN_0938 | 1 | 1 | 1 | 1 | 1 | 1 |
| FTN_0939 | 1 | 1 | 1 | 1 | 1 | 1 |
| FTN_0965 | 1 | 1 | 1 | 1 | 1 | 1 |
| FTN_1017 | 1 | 1 | 1 | 1 | 1 | 1 |
| FTN_1078 | 1 | 1 | 1 | 1 | 1 | 1 |
| FTN_1102 | 1 | 1 | 1 | 1 | 1 | 1 |
| FTN_1153 | 1 | 1 | 1 | 1 | 1 | 1 |
| FTN_1203 | 1 | 1 | 1 | 1 | 1 | 1 |
| FTN_1204 | 1 | 1 | 1 | 1 | 1 | 1 |
| FTN_1205 | 1 | 1 | 1 | 1 | 1 | 1 |
| FTN_1206 | 1 | 1 | 1 | 1 | 1 | 1 |
| FTN_1207 | 1 | 1 | 1 | 1 | 1 | 1 |
| FTN_1215 | 1 | 1 | 1 | 1 | 1 | 1 |
| FTN_1216 | 1 | 1 | 1 | 1 | 1 | 1 |
| FTN_1304 | 1 | 1 | 1 | 1 | 1 | 1 |
| FTN_1305 | 1 | 1 | 1 | 1 | 1 | 1 |
| FTN_1306 | 1 | 1 | 1 | 1 | 1 | 1 |
| FTN_1307 | 1 | 1 | 1 | 1 | 1 | 1 |
| FTN_1308 | 1 | 1 | 1 | 1 | 1 | 1 |
| FTN_1326 | 1 | 1 | 1 | 1 | 1 | 1 |
| FTN_1327 | 1 | 1 | 1 | 1 | 1 | 1 |
| FTN_1364 | 1 | 1 | 1 | 1 | 1 | 1 |
| FTN_1365 | 1 | 1 | 1 | 1 | 1 | 1 |
| FTN_1366 | 1 | 1 | 1 | 1 | 1 | 1 |
| FTN_1375 | 1 | 1 | 1 | 1 | 1 | 1 |
| FTN_1378 | 1 | 1 | 1 | 1 | 1 | 1 |
| FTN_1379 | 1 | 1 | 1 | 1 | 1 | 1 |
| FTN_1394 | 1 | 1 | 1 | 1 | 1 | 1 |
| FTN_1395 | 1 | 1 | 1 | 1 | 1 | 1 |
| FTN_1396 | 1 | 1 | 1 | 1 | 1 | 1 |
| FTN_1397 | 1 | 1 | 1 | 1 | 1 | 1 |
| FTN_1411 | 1 | 1 | 1 | 1 | 1 | 1 |
| FTN_1419 | 1 | 1 | 1 | 1 | 1 | 1 |
| FTN_1420 | 1 | 1 | 1 | 1 | 1 | 1 |
| FTN_1424 | 1 | 1 | 1 | 1 | 1 | 1 |
| FTN_1428 | 1 | 1 | 1 | 1 | 1 | 1 |
| FTN_1473 | 1 | 1 | 1 | 1 | 1 | 1 |
| FTN_1488 | 1 | 1 | 1 | 1 | 1 | 1 |
| FTN_1489 | 1 | 1 | 1 | 1 | 1 | 1 |
| FTN_1490 | 1 | 1 | 1 | 1 | 1 | 1 |
| FTN_1497 | 1 | 1 | 1 | 1 | 1 | 1 |
| FTN_1498 | 1 | 1 | 1 | 1 | 1 | 1 |
| FTN_1541 | 1 | 1 | 1 | 1 | 1 | 1 |
| FTN_1575 | 1 | 1 | 1 | 1 | 1 | 1 |
| FTN_1577 | 1 | 1 | 1 | 1 | 1 | 1 |
| FTN_1578 | 1 | 1 | 1 | 1 | 1 | 1 |
| FTN_1579 | 1 | 1 | 1 | 1 | 1 | 1 |
| FTN_1614 | 1 | 1 | 1 | 1 | 1 | 1 |
| FTN_1629 | 1 | 1 | 1 | 1 | 1 | 1 |
| FTN_1663 | 1 | 1 | 1 | 1 | 1 | 1 |
| FTN_1698 | 1 | 1 | 1 | 1 | 1 | 1 |
| FTN_1720 | 1 | 1 | 1 | 1 | 1 | 1 |
| FTN_1721 | 1 | 1 | 1 | 1 | 1 | 1 |
| FTN_1722 | 1 | 1 | 1 | 1 | 1 | 1 |
| FTN_1723 | 1 | 1 | 1 | 1 | 1 | 1 |
| FTN_1724 | 1 | 1 | 1 | 1 | 1 | 1 |
| FTN_1727 | 1 | 1 | 1 | 1 | 1 | 1 |
| FTN_1728 | 1 | 1 | 1 | 1 | 1 | 1 |
| FTN_1729 | 1 | 1 | 1 | 1 | 1 | 1 |
| FTN_1731 | 1 | 1 | 1 | 1 | 1 | 1 |
| FTN_1746 | 1 | 1 | 1 | 1 | 1 | 1 |
| FTN_1756 | 1 | 1 | 1 | 1 | 1 | 1 |
| FTN_1758 | 1 | 1 | 1 | 1 | 1 | 1 |
| FTN_1759 | 1 | 1 | 1 | 1 | 1 | 1 |
| FTN_1760 | 1 | 1 | 1 | 1 | 1 | 1 |
| FTN_1761 | 1 | 1 | 1 | 1 | 1 | 1 |
| FTN_1766 | 1 | 1 | 1 | 1 | 1 | 1 |
| FTN_1780 | 1 | 1 | 1 | 1 | 1 | 1 |
| FTN_1781 | 1 | 1 | 1 | 1 | 1 | 1 |

^a^*F. tularensis* subsp. *mediasiatica* FSC147 (GenBank acc. no. CP000915), ^b^*F. tularensis* subsp. *tularensis* A1 SCHUS4 (acc. AJ749949), ^c^*F. tularensis* subsp. *tularensis* A2 WY96-3418 (acc. CP000608), ^d^ *F.tularensis* subsp. *holarctica* LVS (acc. AM233362), ^e^*F. tularensis* subsp. *holarctica* FTA (acc. CP000803), ^f^*F. tularensis* subsp. *holarctica* OSU18 (acc.CP000437).
