# Supplementary material for: Molecular Evolutionary Consequences of Niche Restriction in Francisella tularensis, a Facultative Intracellular Pathogen
Source: PLoS Pathog. 2009 Jun 12;5(6):e1000472. doi: 10.1371/journal.ppat.1000472 (PMC2688086; doi:10.1371/journal.ppat.1000472)
Supplement: Text S1 — Preparation of genomic DNA. (0.03 MB DOC) [file ppat.1000472.s009.doc]

# Materials and Methods, Supporting information

Preparation of genomic DNA

DNA for genome sequencing was prepared at the Swedish Defence Research Agency in a Biosafety level 3 laboratory. Cultures of *F. tularensis* subsp. *mediaiatica* strain FSC147 were incubated for 48 h on GCII agar with 1% hemoglobin and 1% IsoVitaleX at 37 °C in a 5 % CO_2_ humid atmosphere. Bacterial cells were suspended in phosphate buffered saline, pH 7.4, and pelleted. After resuspension in 100 µl 10 mM TRIS-HCl, pH 8.0, with 1 mM EDTA, 5 µl of 100 mg/ml lysozyme was added before incubation at 37°C for 30 minutes. Then, 600ul of Qiagen Buffer ATL (Qiagen), SDS to a final concentration of 0.5% (w/v), and proteinase K were added before incubation at 55°C for 2.5 hours, Preparations were sterility-checked at this point and the preparation was then finalized following a standard protocol including RNAase treatment, phenol-chloroform extractions, and use of Phase Lock Gel Heavy tubes (Eppendorf AG). Retention of the virulence of strain FSC147 was confirmed prior to sequencing. Subcutaneous infection of five mice with <50 cells killed all the mice within four days.
